# A Target Class Ligandability Evaluation of WD40 Repeat-Containing Proteins

**DOI:** 10.1021/acs.jmedchem.4c02010

**Published:** 2024-11-04

**Authors:** Suzanne Ackloo, Fengling Li, Magda Szewczyk, Almagul Seitova, Peter Loppnau, Hong Zeng, Jin Xu, Shabbir Ahmad, Yelena A Arnautova, A. J. Baghaie, Serap Beldar, Albina Bolotokova, Paolo A. Centrella, Irene Chau, Matthew A. Clark, John W. Cuozzo, Saba Dehghani-Tafti, Jeremy S. Disch, Aiping Dong, Antoine Dumas, Jianwen A. Feng, Pegah Ghiabi, Elisa Gibson, Justin Gilmer, Brian Goldman, Stuart R Green, Marie-Aude Guié, John P. Guilinger, Nathan Harms, Oleksandra Herasymenko, Scott Houliston, Ashley Hutchinson, Steven Kearnes, Anthony D. Keefe, Serah W. Kimani, Trevor Kramer, Maria Kutera, Haejin A. Kwak, Cristina Lento, Yanjun Li, Jenny Liu, Joachim Loup, Raquel A. C. Machado, Christopher J. Mulhern, Sumera Perveen, Germanna L. Righetto, Patrick Riley, Suman Shrestha, Eric A. Sigel, Madhushika Silva, Michael D. Sintchak, Belinda L. Slakman, Rhys D. Taylor, James Thompson, Wen Torng, Carl Underkoffler, Moritz von Rechenberg, Ryan T. Walsh, Ian Watson, Derek J. Wilson, Esther Wolf, Manisha Yadav, Aliakbar K. Yazdi, Junyi Zhang, Ying Zhang, Vijayaratnam Santhakumar, Aled M. Edwards, Dalia Barsyte-Lovejoy, Matthieu Schapira, Peter J. Brown, Levon Halabelian, Cheryl H. Arrowsmith

**Affiliations:** 1 Structural Genomics Consortium, University of Toronto, 101 College St., Toronto, ON M5G 1L7, Canada; 2 Google, 1600 Amphitheatre Parkway, Mountain View, California 94043, United States; 3 ZebiAI Inc., 100 Beaver St., Waltham, Massachusetts 02435, United States; 4 X-Chem Inc., 100 Beaver St., Waltham, Massachusetts 02435, United States; 5 Relay Therapeutics, 399 Binney St., Cambridge, Massachusetts 02139, United States; 6 Princess Margaret Cancer Centre, University of Toronto, Toronto, ON M5G 2M9, Canada; 7 Civetta Therapeutics, 10 Wilson Rd., Cambridge, Massachusetts 02138, United States; 8 Department of Chemistry, York University, Toronto, ON M3J 1P3, Canada; 9 Department of Chemistry, University of Toronto, Toronto, ON M5S 3H6, Canada; 10 Department of Pharmacology and Toxicology, University of Toronto, Toronto, ON M5S 1A8, Canada

## Abstract

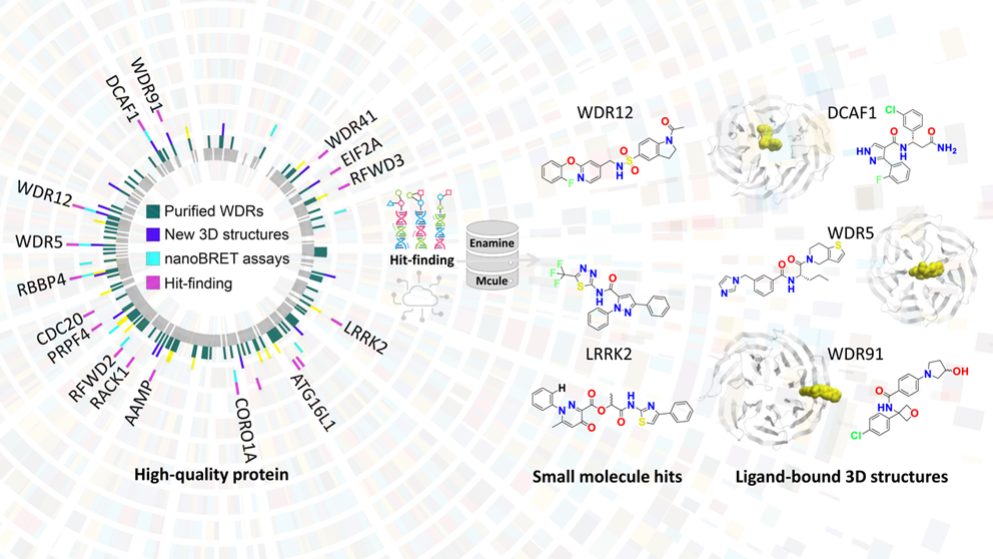

Target class-focused drug discovery has a strong track record in pharmaceutical research, yet public domain data indicate that many members of protein families remain unliganded. Here we present a systematic approach to scale up the discovery and characterization of small molecule ligands for the WD40 repeat (WDR) protein family. We developed a comprehensive suite of protocols for protein production, crystallography, and biophysical, biochemical, and cellular assays. A pilot hit-finding campaign using DNA-encoded chemical library selection followed by machine learning (DEL-ML) to predict ligands from virtual libraries yielded first-in-class, drug-like ligands for 7 of the 16 WDR domains screened, thus demonstrating the broader ligandability of WDRs. This study establishes a template for evaluation of protein family wide ligandability and provides an extensive resource of WDR protein biochemical and chemical tools, knowledge, and protocols to discover potential therapeutics for this highly disease-relevant, but underexplored target class.

## Introduction

WD40 Repeat (WDR) proteins comprise one of the largest protein families, with ∼349 WDR encoding genes in the human genome. WDRs are generally understudied (Figure S1a, Table S1) though many have strong genetic links to human diseases and are involved in a wide variety of cellular processes including epigenetics, ubiquitin signaling, DNA repair, RNA splicing, and immune system signaling.^1^ This family harbors more “essential genes” in cancer than any other protein family (Figure S1b, Table S1),^2^ and is also involved in many neurological and inflammatory diseases. Despite the strong links to disease and unlike other large target classes such as GPCRs or kinases, the WDR family is largely unexplored with respect to drug discovery.

**Figure 1:**
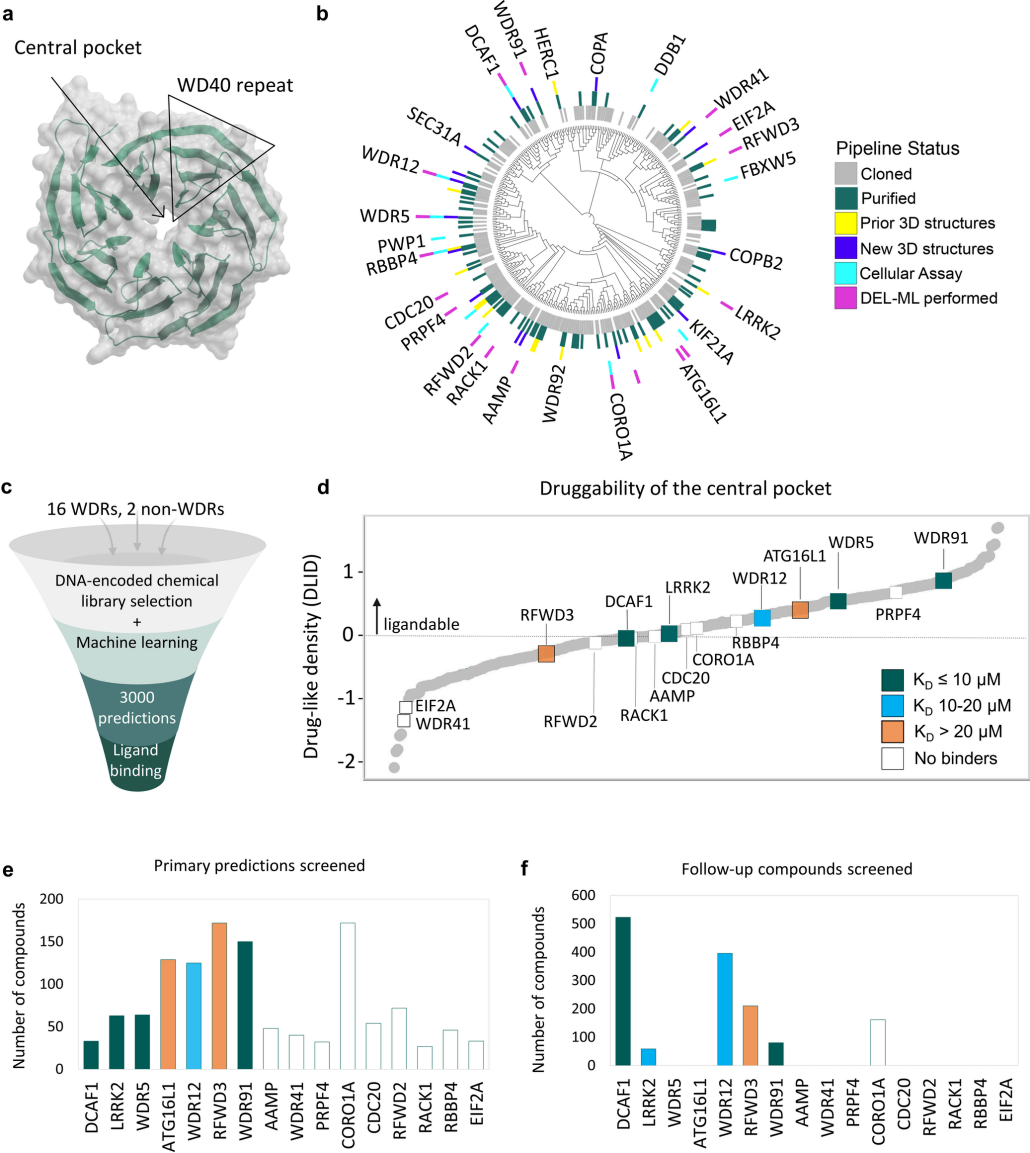
A roadmap for developing target-class focused pharmacological tools. (**a**) Structure of a canonical 7-bladed WDR protein (PDB ID: 7KQQ) is shown in ribbon and surface representations. The triangle highlights one WD40 repeat, and the arrow points to the central pocket. (**b**) A phylogenetic tree of the WDR protein family with annotations for targets as follows: cloned (grey), purified (green), new 3D structures (blue), prior 3D structures (yellow), cellular target engagement assays (cyan), and subjected to hit-finding (pink) in this study. (**c**) The hit-finding funnel encompassing DEL selections (Table S9) to ligand binding assays (Table S4). (**d**) Ligandability of the central pocket in AlphaFold-generated structures^31^ evaluated with the drug-like density index (DLID)^35^ calculated with ICM (Molsoft, San Diego) across all human WDR containing proteins where pockets predicted to be ligandable have a DLID > 0; details in Table S8. The WDRs are color-coded based on the most potent hit discovered by DEL-ML. (**e**) The number of primary predicted ligands tested, and (**f**) the number of follow-up compounds tested. The follow-up compounds were selected and prioritized using the same GCNN model that was used to predict the primary hits. Follow-up compounds were ordered for DCAF1, LRRK2, WDR12, RFWD3, and WDR91. (WDR12 is annotated with an light blue in (f) because MR44915 (*K*_D_ value of 4 μM) was discovered by medicinal chemistry optimization of MR40903.) In (d–f): targets with a hit having *K*_D_ value ≤ 10 μM are indicated with green, targets with hits having *K*_D_ values between 11 and 20 μM are indicated with light blue, targets with hits having *K*_D_ values > 20 μM are indicated with orange, and targets with no hits are indicated with no color.

The WDR domain is a protein–protein interaction (PPI) module often acting as a substrate recognition or scaffolding subunit within larger multiprotein complexes.^1,3,4^ WDR domains all have a canonical donut shape comprising 7–9 β-propellers; each β-propeller contains the namesake 40-residue WD (tryptophan-aspartate) repeat sequence (Figure 1a). Although WDRs have been shown to mediate PPIs through many surfaces of their conserved fold, the central pocket is frequently involved in key functional interactions with peptide regions of partner proteins such as degrons or the recognition of post-translational modifications. Furthermore, some WDRs also bind nucleic acids.^4,5^

Interestingly, neither the outer surface of the WDR donut, nor the residues lining the central pocket are conserved across the WDR family (Table S2, Figure S1c). With the exception of a few very closely related subfamilies, e.g., the coronins, plexins, and semaphorins (Figure S1c), WDRs are only conserved in their protein fold but not in their functional interaction sites. Nevertheless, the central pocket often has the appropriate size and physicochemical properties for potent binding to drug-like small molecules. We were among the first to demonstrate that it is possible to disrupt PPIs of WDR proteins with drug-like small molecules that compete with or enhance binding of native interacting proteins or peptides. For example, antagonists of WDR5 can disrupt its interaction with Mixed Lineage Leukemia (MLL) histone methyltransferase (Figure S2a), thereby abrogating MLL function to suppress growth in leukemia and breast cancer.^6−9^ Similarly, small molecule antagonism of the central pocket of EED (Figure S2b), a WDR subunit of the polycomb repression complex 2 (PRC2), disrupts binding of PRC2-stimulatory proteins with concomitant inhibition of PRC2 catalytic activity.^10,11^

There is growing interest in the ligand discovery for WDRs from the context of degrader modalities. For example, DCAF1^12−14^ and DCAF11^15^ are substrate recognition subunits and DDB1 is an adaptor protein for the Cullin-RING ubiquitin ligase complex.^16,17^ WDR ligands can also act as activators, for example by “locking in” a native binding partner via a molecular glue mechanism such as for BTRC E3 ligase and β-Catenin^18^ (Figure S2c). Small molecule ligands have also been reported to bind (in pockets) on the outer surface of some WDRs, such as the CDC20 E3 ligase (Figure S2d), but such ligands are of lower potency.^19^

Notwithstanding these exciting results over the past decade or so, the vast majority of WDR proteins are both understudied in terms of their biological function (Figure S1a, b), and unexplored with respect to small molecule antagonism. We hypothesized that the WDR protein family harbors many more druggable members, and set out to test this in the context of Target 2035, a global initiative to develop pharmacological tools for all human proteins.^20−23^ We were particularly interested in developing scalable hit-finding approaches that could be applied broadly to other target families. To this end, we chose DEL-ML,^24^ a high-throughput screening strategy proven for several well-validated target classes,^25−29^ which overcomes many practical liabilities of off-DNA synthesis of “traditional” DEL selection approaches.^30^

Our results indicate that many understudied WDR proteins are indeed ligandable with drug-like small molecules and that DEL-ML has broad applicability across the diverse, nonconserved WDR binding pockets (Figure 1c, Table S2). Our data, methods and related reagents comprise a significant resource to the community that will enable the development of pharmacological tools for WDR proteins to further explore their biology, interaction networks, and therapeutic potential.

## Results and Discussion

### WDRs Readily Purify at Scale with Crystallization-Level Quality

To choose a test set of WDR proteins, we considered proteins that were both strongly linked to diseases such as cancer (Figure S1b), as well as those that were not well characterized, including many for which recombinant protein or experimentally determined 3D structures have not been reported (Table S1). A total of 259 WDR proteins—more than half the 349-member WDR family—were selected for recombinant expression and purification^31^ (Tables S3, S4).

Previous experience in our group suggested that recombinant expression of human WDR domains in a Baculovirus Expression Vector System (BEVS) was often more successful than expression in *E. coli*. We therefore designed, on average, two expression constructs for each of the WDRs and subcloned these for baculovirus-mediated expression in Sf9 cells. Because of conservation of the overall WDR fold, we were able to readily predict the domain boundaries, including the frequent addition of helices or small loops and folded regions internally or at the immediate termini, using homology modeling; this initial phase of the project predated the release of AlphaFold.^32−34^ Of the 259 proteins tested for expression, 95 were purified at scale with at least one affinity tag and 24 with a luminescent tag for cellular target engagement assays (Figure 1b, Tables S3, S4).

Quality control of the recombinant protein included using SDS-PAGE and mass spectrometry to confirm purity (>90%) and the expected molecular weight, respectively. Finally, differential scanning fluorimetry (DSF) (Table S5) was performed to ensure that each purified protein was stable and folded in the absence of its interaction partner(s). These data showed that a large proportion of WDR domains can be produced in isolation for further study—including many that are central components of large macromolecular complexes. Protein crystallization was attempted for select WDRs that had sufficient yields to set up at least one 96-well crystallization tray and homogeneous elution profiles on size exclusion chromatography. Overall, 14 apo WDR structures were generated to support structure-guided drug discovery efforts (Figure 1b, Tables S6, S7). The protocols for expressing, purifying and crystallizing the apo WDR domains greatly facilitated the rapid characterization of ligands.^31^

Upon release of the AlphaFold structure database,^33^ we analyzed the electrostatics and central pocket ligandability of all 349 human WDR-containing proteins with predicted structures. The use of AlphaFold predictions to map electrostatics and ligandable pockets in WDRs was supported by our observation that less than 1.3 Å backbone RMSD separates the predicted WDR structures of UTP15 or EIF2A and their subsequently released crystal structures (PDB IDs: 7RUO, 8DYS, Tables S7c, S7d).^31^ Overall, about half of the central pockets were predicted to be ligandable^35^ (drug-like density (DLID) > 0, Figure 1d, Table S8), which we believe is a conservative estimate considering that pockets are sometimes occluded in the absence of ligands. For example, the central pocket of the apo structure of EED is occluded by several side chains which are displaced by binding of drug-like small molecule ligands.^10^ The diversity in shape and electrostatics of the central pocket was striking.^31,36^ In fact, it was previously shown that structural diversity is observed even between two WDR binding sites targeted by the same endogenous ligand. For example, an arginine occupies the structurally dissimilar central pockets of the epigenetic regulators WDR5 and RBBP4^3^ yet WDR5 ligands do not bind to RBBP4. This analysis suggests that the discovery of selective ligands for a given WDR protein of interest is quite feasible.

### Many WDRs Are Ligandable

In order to assess WDR domain ligandability, we sought a methodology that could be readily implemented across the WDR family with high throughput and under approximately the same conditions. We chose DEL-ML, originally described by McCloskey and colleagues^24^ and recently by others,^25−27,29^ as it makes use of our high-quality WDR proteins and avoids the need for off-DNA synthetic chemistry post-selection. We selected 15 N-terminally biotin- or his-tagged WDRs that had no reported small molecule ligands at the start of this program (Figure 1b). We also included WDR5 as a positive (family class) control, as it is druggable and has elicited clinical interest in the context of treating leukemias.^6,8,37^ For comparison we also included two non-WDR, methyllysine binding domains known to be ligandable; the PWWP^38,39^ domain of DNMT3A and the triple Tudor domain (TTD) of SETDB1.^40,41^

### Overview of the Hit-Finding Workflow

The DEL deck used for each of the screens reported here comprised between nineteen and sixty-five libraries depending upon the screen. Each library was synthesized independently by applying a split-and-pool methodology to enact a specific chemical scheme with a specific set of building blocks.^30^ This approach allows many thousands of building blocks to be reacted with each other in a combinatorial manner to access multiple billions of diverse and distinct compounds.^30^ This diverse set of encoded compounds comprises common structural motifs optimized for small molecule drug discovery. Screening of the DEL deck was accomplished by the affinity-mediated selection of a mixture of libraries. Captured targets and associated library members were stringently washed under native conditions. This was followed by the recovery of retained library members by the thermal denaturation of the target. After two cycles of selection and recovery, library members’ DNA tags were amplified by PCR and sequenced to generate a minimum of one million reads per library per condition.

After parallel selection outputs were sequenced, normalized, amplification duplicates removed, sequence identifiers translated back into chemical and condition identifiers, and the statistical significance of all building block combinations calculated in all conditions, the quantity and quality of the enriched compounds was assessed. Featurized structural information of enriched building block combinations resulting from each of these selections formed the Positive Training Examples (PTEs) and input for training and evaluating machine-learned models^24^ (Figure S3a, Tables S9, S10). Trained models were then used to predict the likelihood of binding to commercially available compounds from large, made-on-demand, or physical libraries such as Enamine REAL^42^ and Mcule^43^ databases. Top scoring compounds were selected by a directed sphere exclusion clustering method to generate a diverse set of molecules to purchase. All predictions comprise chemical templates that are readily amenable to structure–activity exploration and optimization (Table S11).

For each of the 16 WDRs, between 33 and 200 predicted compounds were purchased depending on the number of overall predictions for the specific target, the ability of the predictions to be synthesized with > 90% purity, and in-stock availability (Figures 1e, f, Table S11). Thus, a total of ∼3000 compounds were purchased for testing in orthogonal assays. Predicted ligands for an additional two targets (CDC40 and SMU1) were not purchased, although their SMILES are listed in Table S11 as a reference. All hit-finding statistics are based on 16 WDRs (Figure 1d–f).

### Ligand Profiling and Binding Characterization

Prior to binding assays, each compound was evaluated for solubility and aggregation up to 200 μM by dynamic light scattering (DLS),^44−46^ to identify potential sources of assay interference. Soluble compounds were assayed for binding to their predicted targets using a variety of biophysical and biochemical methods. For this, we chose surface plasmon resonance (SPR) as the primary assay for hit validation, as it is a generic, well-developed method for detecting small-molecule interactions with a protein immobilized on a biosensor chip via an affinity tag. We found that most WDRs are readily assayed by SPR under a limited set of standard buffer conditions (Table S5). SPR has good throughput and can be run in screening mode for many compounds or in dose–response mode to determine the affinity of individual compounds (Table S11).

In a similar approach to McCloskey and colleagues,^24^ we compared the extent to which the predictions are structurally similar to the PTEs (Figure S3b). Fingerprint similarities were calculated using 1024-bit ECFP6 fingerprints, and Tanimoto distances were calculated between the predictions and PTEs used to train each model. Fingerprint similarities are reported in Table S11 for the most similar PTE to each prediction, as well as to the average fingerprint similarity of the top ten most similar PTEs to the prediction. As observed by McCloskey et al.,^24^ the most potent hits (annotated by a plus sign in Figure S3b) are structurally dissimilar relative to the most similar PTE. Among the hits discovered in this study, only the primary hit for DCAF1 is structurally similar (with a Tanimoto similarity of 0.81) to the closest PTE.

We subsequently employed X-ray crystallography to characterize the binding site of protein–ligand complexes (Tables S6, S7). In some cases, the binding site of the ligand could also be identified by a peptide displacement assay using fluorescence polarization (FP), or hydrogen–deuterium exchange mass spectrometry (HDX-MS).^47^ In the remaining sections, we discuss each hit discovered by DEL-ML, and then evaluate the submicromolar hit for WDR5 in nanoBRET^48^ and CETSA^49^ cellular target engagement assays (Tables S12, S13).

### Hit Expansion of a Primary Hit for DCAF1

The primary hit discovered in this study and its medicinal chemistry optimization to a nanomolar ligand has been published.^50^ Independently, SAR optimization was attempted by using computational methods. The GCNN model that was used to select the primary set of ligands (Figure 1e) was used to search for, and prioritize analogs in virtual libraries. An additional 547 compounds were purchased (annotated as Round 2 in Table S11) and tested. Eighteen compounds bind with *K*_D_ values ≤ 50 μM, and 14 are discussed here (Table 1).

**Table 1:**
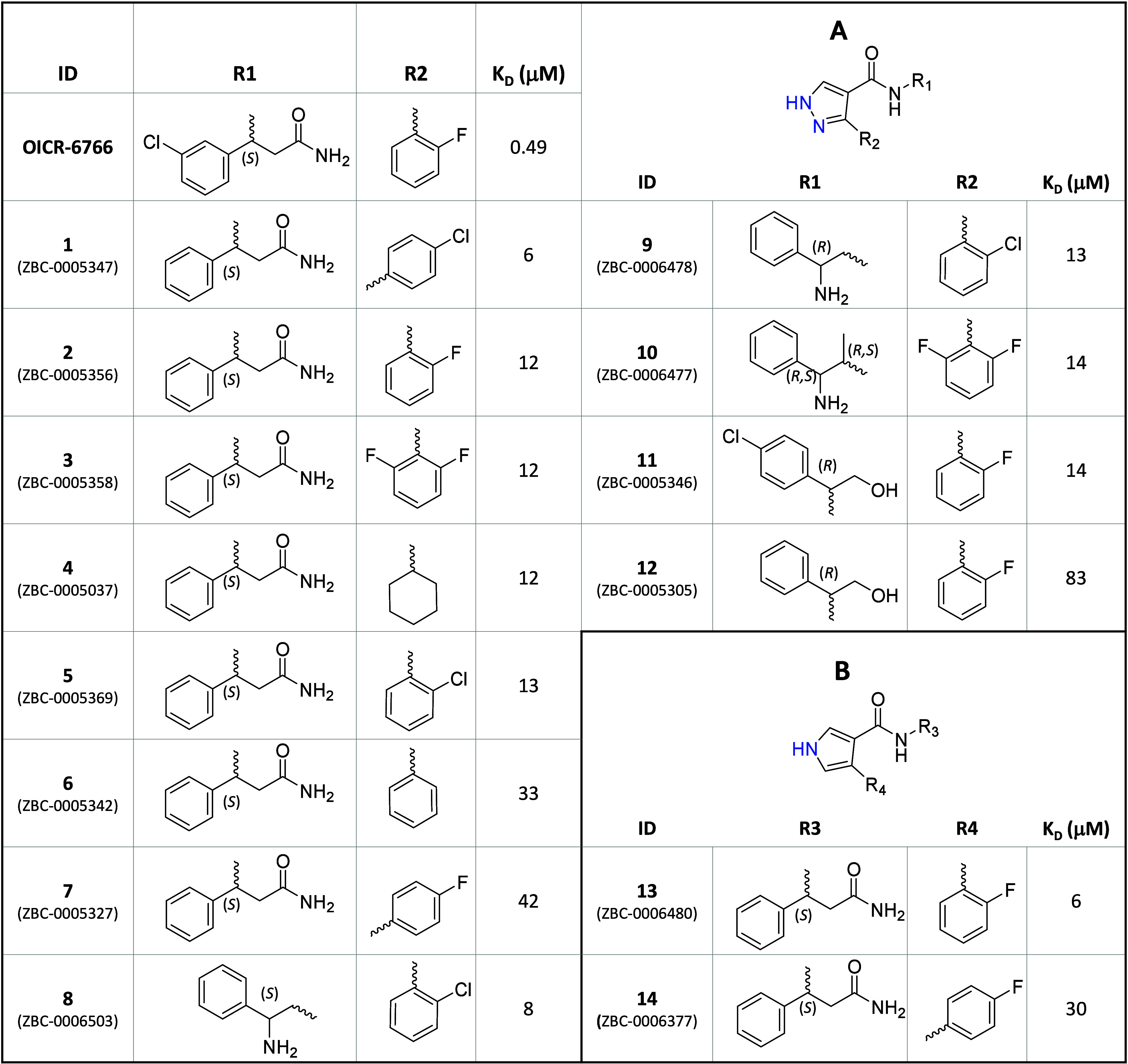
Highlights of the SAR Gleaned from Computational Hit Expansion of a Primary Hit for DCAF1^a^

a Analogs from two cores are listed and the respective stereocenter is annotated in the R1 and R3 columns. (The names in parentheses are those listed in Table S11.)

The (*S*)-enantiomer of phenylpropanamide (compounds **1**–**5**) tolerates several R2 moieties. For example, *para*-chlorophenyl (**1**), *ortho*-fluorophenyl (**2**), 2,6-difluorophenyl (**3**), cyclohexyl (**4**), and *ortho*-chlorophenyl (**5**) are relatively equipotent. However, unmodified phenyl (**6**) and *para*-fluorophenyl (**7**) are not well-tolerated. Propanamide in R1 (e.g., **2**, **3**, **5**) may be replaced by two- or three-carbon amines with either chirality (**8**–**10**), but can only be replaced by ethanol (**11**) when in combination with *para*-chlorophenyl in R1 (**11** relative to **12**). The core pyrazole (**A**) may be replaced by pyrrole (**B**), e.g., **3** and **13**, but both cores better tolerate R2 with *ortho*- (2, 13) rather than *para*-fluorophenyl (**7**, **14**).

Interestingly, contemporaneous with our studies,^50^ novel ligands for DCAF1 were discovered using computational screening methods^51^ and HTS.^13^ Both reported ligands bind closer to the outer surface of the central pocket (PDB ID: 7SSE, 8OGA) and taken together with these results indicate that DCAF1 is a fairly druggable WDR.

### WDR91 Small Molecule Ligands Bind in a Unique Pocket Relative to a Native Binding Partner RAB7A

DEL-ML hit-finding for WDR91 led to the discovery of MR45279 with a *K*_D_ value of 6 μM (PDB ID: 8SHJ), and subsequently to a covalent analog MR46654 (PDB ID: 8T55).^52^ Unlike the hits discovered for DCAF1,^50^ MR45279 and MR46654 bind in a side pocket nestled between two β-propeller blades located at the bottom face of the WDR domain (Figure S4). A recent structure (PDB ID: 8KB8) of WDR91 and GTP-bound RAB7A illustrates that the latter also binds at the bottom face of WDR91 but at a unique site relative to the small molecule ligands (Figure S4).^54^ Importantly, MR45279 demonstrates that side pockets,^3^ which sometimes serve as additional, functional interaction sites in WDRs (Figure S2d), can also be targeted by small molecules with good affinity.

### A Novel Chemotype Was Discovered for the Highly Druggable WDR5

**Figure 2:**
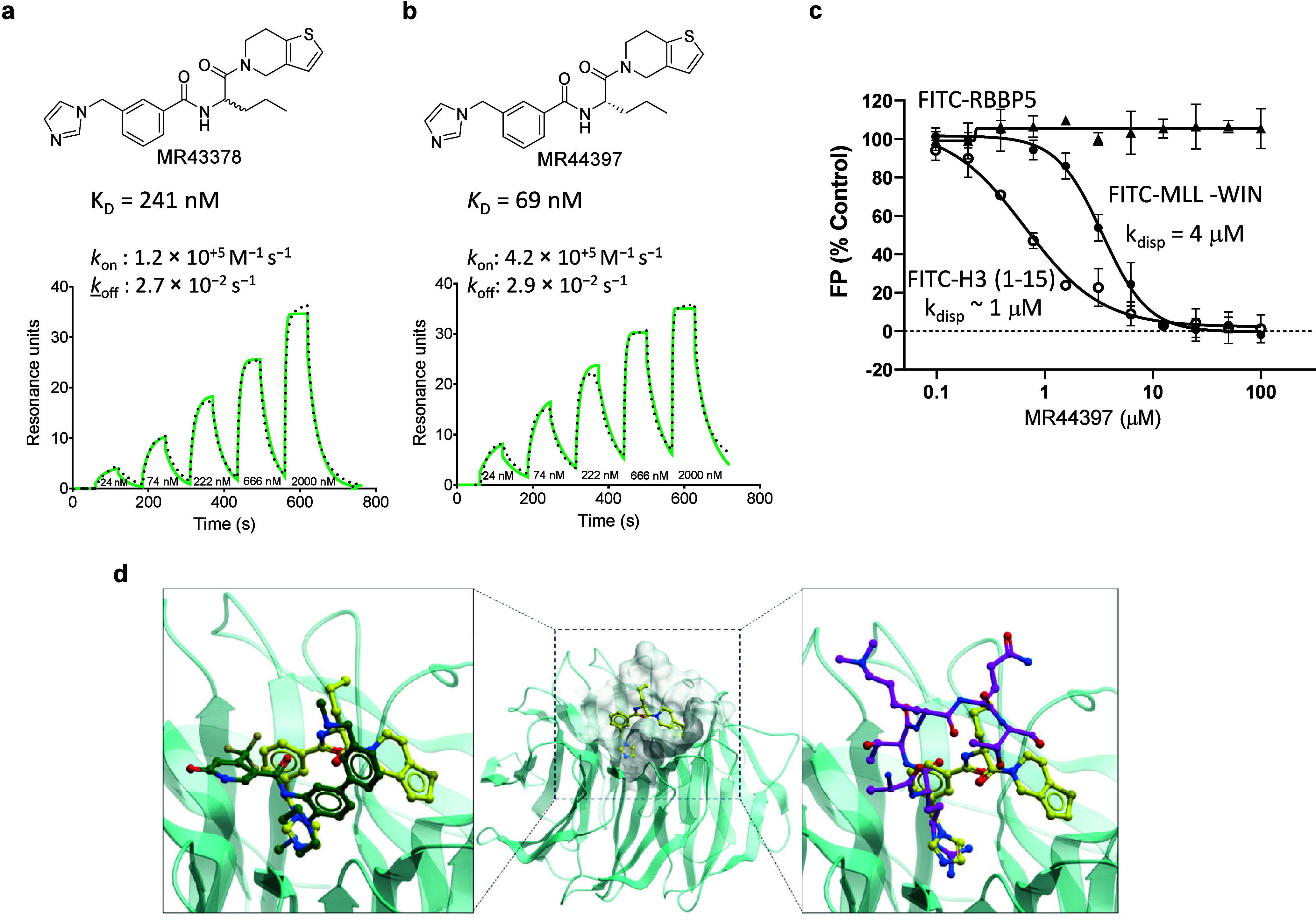
Characterization of a novel WDR5 ligand. (**a**) Chemical structure of primary hit (racemate) MR43378, and an SPR sensorgram showing a dose–response titration for binding to WDR5 with a kinetic fit (green line) to a 1:1 binding model. (**b**) Chemical structure of MR44397 (the (*S*)-enantiomer of MR43378), and an SPR sensorgram showing a dose–response titration for binding to WDR5 with a kinetic fit (green line) to a 1:1 binding model. (**c**) FP-based displacement assays (with WDR5 protein at 5 μM) shows that MR44397 displaces the FITC-H3 (○ ARTKQTARKSTGGKA) with *K*_disp_ = 1 μM, and the FITC-MLL-WIN peptide (● GSARAEVHLRKS) with *K*_disp_ = 4 μM, but does not displace FITC-RBBP5 peptide (▲ EDEEVDVTSV) which binds at a different site. (**d**) (**Center**) Co-crystal structure of WDR5 (cyan) in complex with MR44397 (yellow sticks, PDB ID: 8T5I); (**left**) a close up view of the MR44397 binding site showing superimposed structures of WDR5 (cyan) in complex with MR44397 (yellow sticks) and WDR5 in complex with the potent WDR5 antagonist OICR-9429 (green sticks, PDB ID: 4QL1);^6^ and (**right**) superimposed structures of WDR5 (cyan) in complex with MR44397 (yellow sticks) and WDR5 in complex with a histone H3K4 peptide (magenta sticks, PDB ID: 2O9K).^54^ DSF and BLI data for MR44397 are in Figure S5. The SPR dose–response titration is done from a top concentration of 2 μM with 3-fold dilutions to 24 nM.

Of 64 predicted ligands for WDR5, nine had SPR *K*_D_ values less than 100 μM (Table S11). Two of the most potent compounds are racemates featuring an imidazole and/or tetrahydrobenzothiophene or pyridine. The most potent ligand MR43378 (*K*_D_ value of 241 nM) was subjected to chiral separation and subsequent testing by SPR showed that the *S*-enantiomer (MR44397) had a *K*_D_ value of 69 nM (Figures 2a, b).

A suite of orthogonal assays, including DSF, FP, and BLI, confirmed potent binding (Figures 2, S5). MR44397 substantially stabilized WDR5 as measured by DSF (Figure S5a) and displaced two central pocket-binding peptides (histone H3 and MLL-WIN peptides)^54^ with *K*_disp_ of 1 μM, and 4 μM, respectively (Figure 2c). Importantly, MR44397 did not displace an RBBP5 peptide (aa 371–380), which binds on a surface outside the central pocket of WDR5 (Figure 2c).^55^ To gain structural insights into the binding mode of the compound, we determined the cocrystal structure of MR44397 in complex with WDR5, referred to here as WDR5-MR44397 (Tables S6, S7e). The compound occupies the central pocket of WDR5 (Figure 2d), which is in agreement with the FP displacement data (Figure 2c). These data clearly demonstrate a new chemically tractable series for WDR5, which modulates functional protein interactions of the target.

### WDR12 Ligand Binds at the Top of the Central Pocket

**Figure 3:**
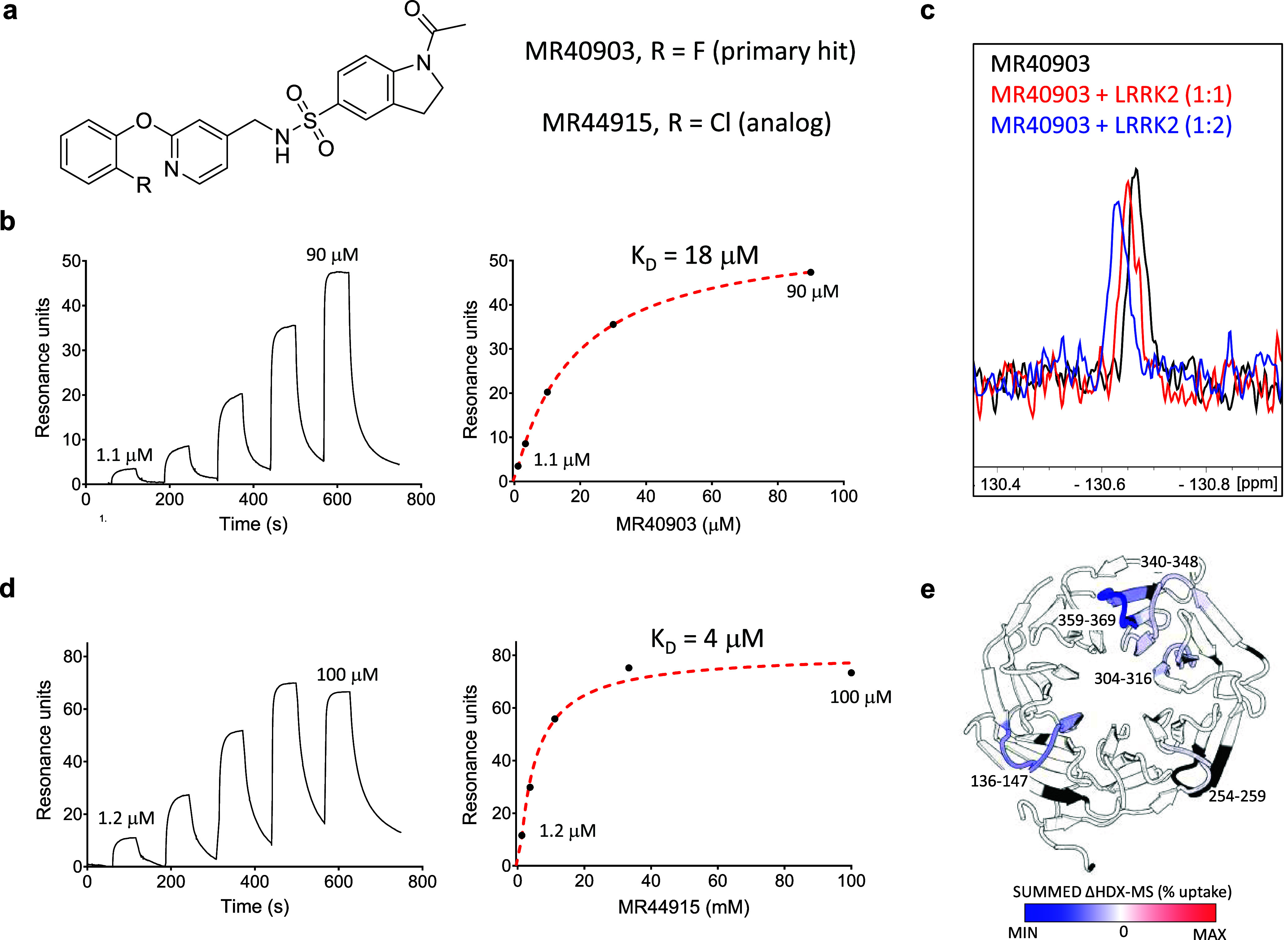
A novel ligand for WDR12 appears to bind near the central pocket. (**a**) Chemical structure of MR40903 (DEL-ML hit) and its chlorine analog MR44915 (Table S11). (**b**) (**Left**) SPR sensorgram showing a dose–response titration (from 90 μM with 3-fold dilutions to 1.1 μM) for MR40903 binding to WDR12, and (**right**) response vs concentration plot using a steady state affinity fit to a 1:1 binding model, *K*_D_ = 18 μM. (**c**) Dose–response, ligand observed ^19^F-NMR for 10 μM of the primary hit MR40903 binding to WDR12, (**d**) (**Left**) SPR sensorgram showing a dose–response titration (from 100 μM with 3-fold dilutions to 1.2 μM) for MR44915 binding to WDR12, and (**right**) response vs concentration plot using a 1:1 steady state affinity fit to a 1:1 binding model, *K*_D_ = 4 μM. (**e**) A cartoon representation of apo WDR12 (PDB: 6N31) with shading representing deuterium uptake in the HDX-MS experiment. The regions shaded in blue have the lowest rate of deuterium uptake. There was no sequence coverage for the regions shaded in black (with additional details in Figure S6).

For WDR12, we tested 530 predicted ligands, for which 39 showed dose–response binding (with *K*_D_ < 100 μM) by SPR. Interestingly, the majority of these binders shared structural features including methyl sulfonamides or methyl sulfones, biaryl or aryl cyclohexyl ethers, providing limited SAR (Table S11). MR40903 is the highest affinity ligand (*K*_D_ value of 18 μM) which harbors a fluorine atom which, when replaced with chlorine yielded MR44915 with slightly better affinity (*K*_D_ value of 4 μM, Figures 3a–c). Dose–response binding of MR40903 was confirmed using ^19^F-NMR (Figure 3d), while HDX-MS^47^ confirmed binding of the more potent MR44915 (Figures 3e, S6). HDX-MS of WDR12 in the presence of MR44915 revealed regions around the central pocket (shaded in blue) that were protected from deuterium uptake. Moreover, these protected regions included three peptides on the “top” of the WDR fold that point toward the central pocket (Figures 3e, S6). These data suggest that MR44915 likely binds near the top of the “donut”.

### Two Chemotypes for LRRK2 Were Confirmed by Ligand Observed ^19^F-NMR

**Figure 4:**
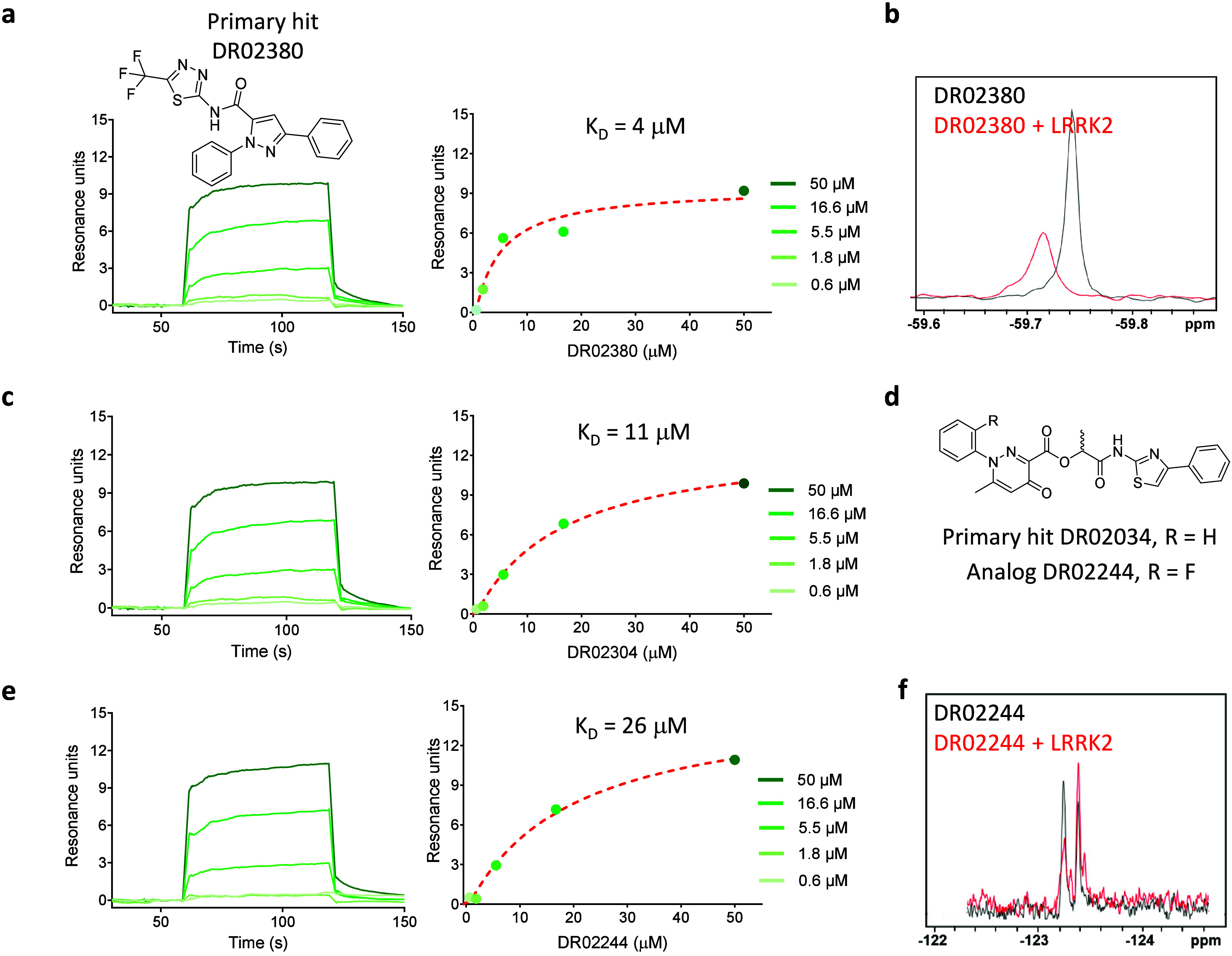
| Two unique chemotypes were discovered for the WDR domain of LRRK2. (**a**) (**Left**) SPR sensorgram showing a dose–response titration for DR02380 binding to LRRK2, and (**right**) response vs concentration plot using a steady state affinity fit to a 1:1 binding model (red, dashed lines), *K*_D_ = 4 μM. (**b**) (Ligand observed) ^19^F-NMR spectrum of 10 μM DR02380 in the absence (black) and presence (red) of 20 μM LRRK2 WDR domain, demonstrating a significant change (in peak height and broadening) in the chemical environment of the trifluoromethyl group. (**c**) (**Left**) SPR sensorgram showing a dose–response titration for DR02034 binding to LRRK2, and (**right**) response vs concentration plot using a steady state affinity fit to a 1:1 binding model (red, dashed lines), *K*_D_ = 11 μM. (**d**) Chemical structures of DR02034 and a fluorine analog DR02244. (**e**) (**Left**) SPR sensorgram showing a dose–response titration for DR02244 binding to LRRK2, and (**right**) response vs concentration plot using a steady state affinity fit to a 1:1 binding model, *K*_D_ = 20 μM. (**f**) (Ligand observed) ^19^F-NMR spectrum of 10 μM DR02244 in the absence (black) and presence (red) of 20 μM LRRK2, demonstrating a small but noticeable change (in intensity and splitting) in the chemical environment of the fluorine atom. The SPR dose–response experiments start at a top concentration of 50 μM with 3-fold dilutions to 0.6 μM.

First-in-class ligands for the WDR domain of LRRK2 were discovered. Of ∼70 predicted compounds tested for LRRK2, two hits with unique chemotypes were identified (Figure 4). Both compounds bind in a dose-dependent manner, but the level of binding is notably less than ideal (Table S11). DR02380 binds to LRRK2 with an estimated *K*_D_ value of 4 μM with 20% binding (Figure 4a, Table S11). Taking advantage of the trifluoromethyl group, we used ^19^F-NMR as an orthogonal method to confirm binding (Figure 4b). The second chemotype, DR02034 (Figures 4c, d), has an estimated *K*_D_ value of 11 μM with ≥30% binding from both the original batch and the repurchased solid, respectively (Table S11). Binding of DR02034 was confirmed using ^19^F NMR and a fluorophenyl analog DR02244 (Figures 4e, f).

### Hit-Finding for the Remaining WDRs

We discovered very weak binders for RFWD3, and ATG16L1 but no binders for nine WDRs. For RFWD3, there were four compounds (from 211 screened) which showed dose–response binding, but only one with a *K*_D_ value < 50 μM (Table S11). For ATG16L1 there was only one compound (out of 129) for which dose–response binding yielded a *K*_D_ value of 44 μM (Table S11). Binding of each compound to their respective target was confirmed from solid material but not by an orthogonal method. The discovery of small molecule binders may indicate some degree of ligandability for both RFWD3 and ATG16L1.

### Histone “Reader” Domains Are Also Amenable to Hit-Finding by DEL-ML

**Figure 5:**
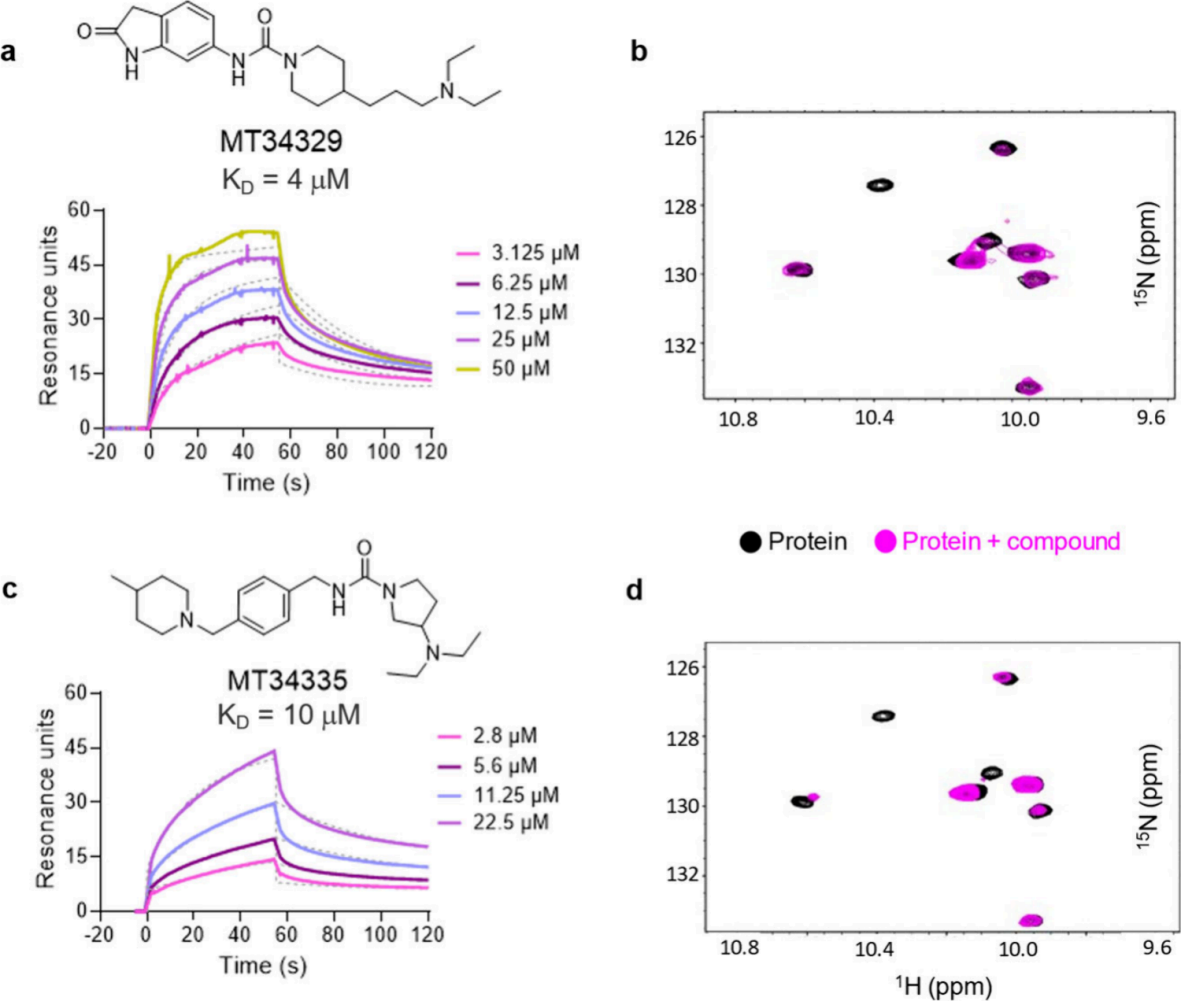
A novel chemotype for DNMT3A is confirmed by NMR. (**a**) Chemical structure of MT34329 and a dose–response SPR sensorgram with a kinetic fit to a 1:1 binding model (**—**) yielded *K*_D_ = 4 μM. (**b**) Indole proton region of the ^1^H-^15^N HSQC spectra of 100 μM ^15^N-labeled DNMT3A-PWWP (●) and ^15^N-labeled DNMT3A-PWWP with 500 μM of MT34329 (●). (**c**) Chemical structure of MT34335 and a dose–response SPR sensorgram with a kinetic fit to a 1:1 binding model (**—**) yielded *K*_D_ = 10 μM. (**d**) Indole proton region of the ^1^H-^15^N HSQC spectra of 100 μM ^15^N-lablelled DNMT3A-PWWP (●) and ^15^N-lablelled DNMT3A-PWWP with 500 μM of MT34335 (●). In the presence of MT34329 and MT34335 we observe general broadening and/or chemical shift perturbations of a common subset of PWWP resonances. The full HSQC spectra are in Figure S7.

We have previously shown that PWWP^38,39^ and tandem Tudor domains are druggable. Further, these PWWP ligands have been used as chemical starting points for degrader modalities.^56,57^ DNMT3A is a DNA methyltransferase that is essential for establishing and maintaining DNA methylation patterns. Its PWWP domain binds methylated histone marks, guiding DNMT3A to specific genomic locations.^58,59^ Of 90 predicted ligands for DNMT3A, 3 have *K*_D_ values < 20 μM. The three ligands have a diethylamino moiety which may mimic methyllysine (Figure 5, Table S11). Binding of MT34329 and MT34335 to DNMT3A-PWWP was confirmed by measuring NMR chemical shift perturbations of amide resonances in ^1^H-^15^N HSQC spectra of a uniformly ^15^N-labeled PWWP domain (Figures 5b, 5d, S7). In the presence of MT34329 or MT34335 (at 5:1 compound to protein ratios), broadening and/or shifting was observed in a subset of the PWWP resonances including several of the tryptophan side chain protons (Figures 5b, 5d).

**Figure 6:**
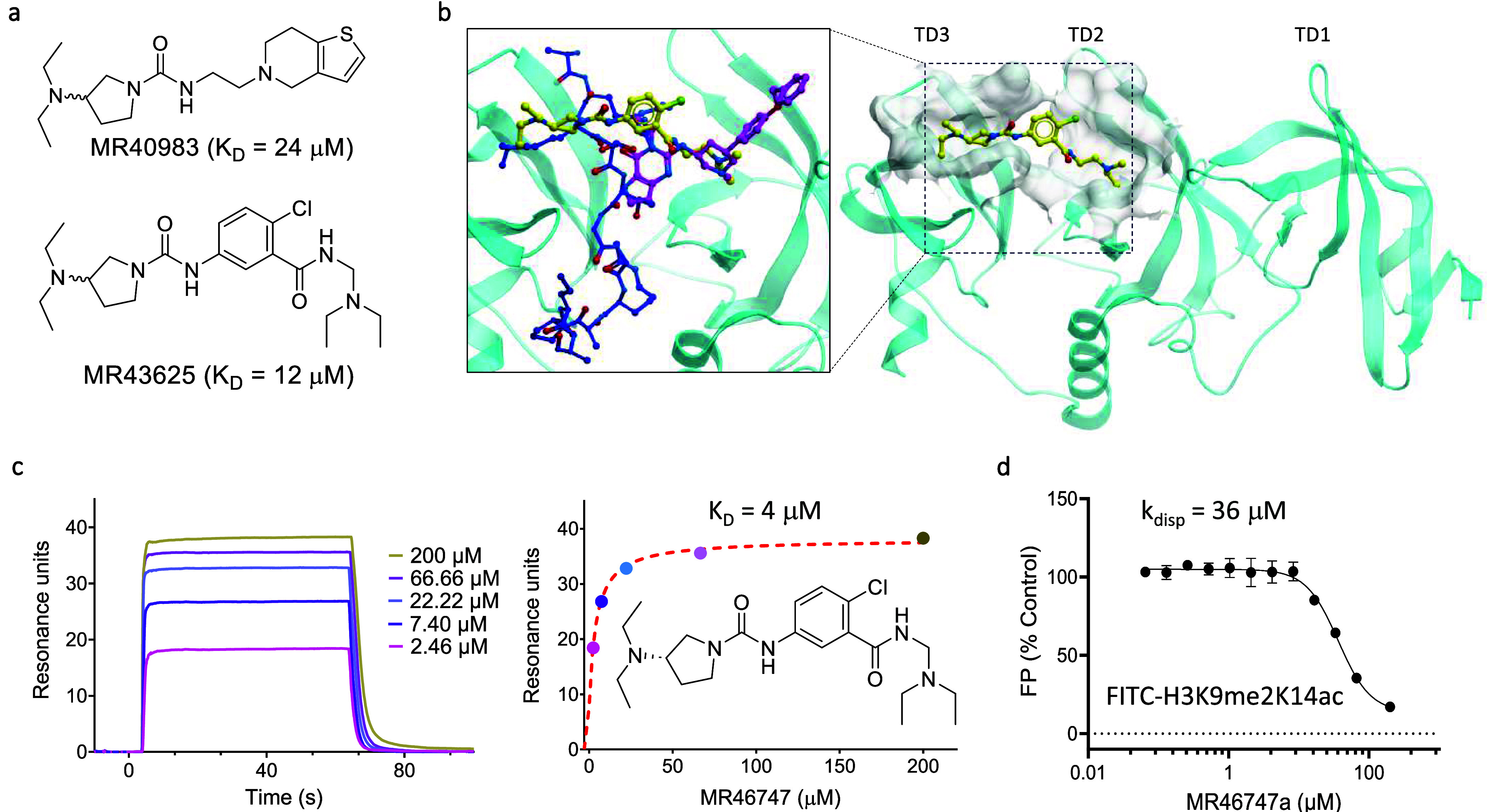
A novel peptide-competitive ligand of SETDB1. (**a**) Chemical structures of racemates MR40983 and MR43625. **(b)** (**Right**) Co-crystal structure of SETDB1-TTD (cyan) in complex with MR46747 (yellow sticks, PDB ID: 8UWP), and (**left**) superimposed crystal structures of SETDB1-TTD (cyan) in complex with MR46747 (yellow sticks, PDB ID: 8UWP), SETDB1-TTD in complex with H3K9me2K14Ac (blue, PDB ID: 6BHD), and SETDB1-TTD in complex with (*R*, *R*)-59 (magenta, PDB ID: 7CJT). The three Tudor domains are indicated as TD1–3. (**c**) (**Left**) SPR sensorgram showing a dose–response titration for MR46747 binding to SETDB1-TTD, and (**right**) the chemical structure of MR46747 (the (*S*)-enantiomer of MR43625) and a response vs concentration plot using a steady state affinity fit (●) and a 1:1 binding model, *K*_D_ = 4 μM. **(d)** MR46747 displaces FITC-H3K9me2K14ac (ARTKQTARK(me2)STGGK(ac)APRKQLATKAA) with *K*_disp_ of 36 μM in an FP-based assay.

The SETDB1 TTD recognizes lysine methylation and acetylation marks on histone H3,^60^ and has several reported ligands.^40,41^ Here we first tested 68 predicted ligands using an FP-based peptide displacement assay. In a displacement assay with FITC-labeled H3K9me2K14Ac, 20 compounds reduced the polarization signal by more than 60% indicating partial displacement (Table S11). These potential ligands were then tested in dose–response by SPR, where six compounds had *K*_D_ values ranging from 24 to 200 μM. The most potent hit is MR40983 (a racemate) with a *K*_D_ value of 24 μM (Figure 6a, Table S11).

Using these preliminary data, compounds were selected from Enamine REAL in an attempt to explore the SAR (Table S11). The resulting set of 107 compounds was tested in a similar approach; first in a peptide displacement assay and then by SPR. Three hits, MR43625, MR43615, MR43579, were identified with the most potent MR43625 (a racemate) having a *K*_D_ value of 12 μM (Figure 6a, Table S11). MR43625 was cocrystallized with SETDB1-TTD (PDB ID: 8UWP) which shows MR43625 binding at the interface between the Tudor domains TD2 and TD3 by inserting one diethylamino moiety into each of the aromatic cages of TD2 and TD3. Further, there appears to be a better fit to the (*S*)-enantiomer (Figure 6b). To confirm this observation, the enantiomers were procured and tested by SPR. MR46747 (the (*S*)-enantiomer of MR43625) binds with a *K*_D_ value of 4 μM (Figure 6c) while the (*R*)-enantiomer of MR43625 binds with a *K*_D_ value of 70 μM (Table S11). Additional profiling shows that MR46747 displaces H3K9me2K14Ac with a *K*_disp_ value of 36 μM (Figure 6d), again demonstrating the discovery of a novel ligand that can displace a native peptide interaction partner.

### The Discovered Hits Are Selective

On average, the members of the WDR protein family have low (≤20%) sequence homology (Table S2, Figure S1c). Nevertheless, we thought that assessing the selectivity would add a valuable dimension to the study. We created a selectivity panel comprising the targets for which we discovered at least one hit with *K*_D_ values ≤ 20 μM. We use SPR to evaluate the level of binding of each hit (at 50 μM) to each protein. Selectivity is reported as normalized percent binding from three SPR experiments.

The hits for WDR5, DCAF1, WDR12, and WDR91 are highly selective for their intended targets (Table 2). The LRRK2 hits are selective when measured in dose–response binding mode (Figure 4) but had consistently lower binding levels (Table S11). This lower percent binding may be due to the apparent poorer overall solubility of these compounds (Table S11). At 50 μM, MT34329 binds to both DNMT3A and DCAF1. However, in a dose–response experiment the affinity of MT34329 for DNMT3A-PWWP (*K*_D_ value of 4 μM, Figure 5a) is 10-fold more potent relative to DCAF1 (*K*_D_ value of 44 μM, Figure S8).

**Table 2:**
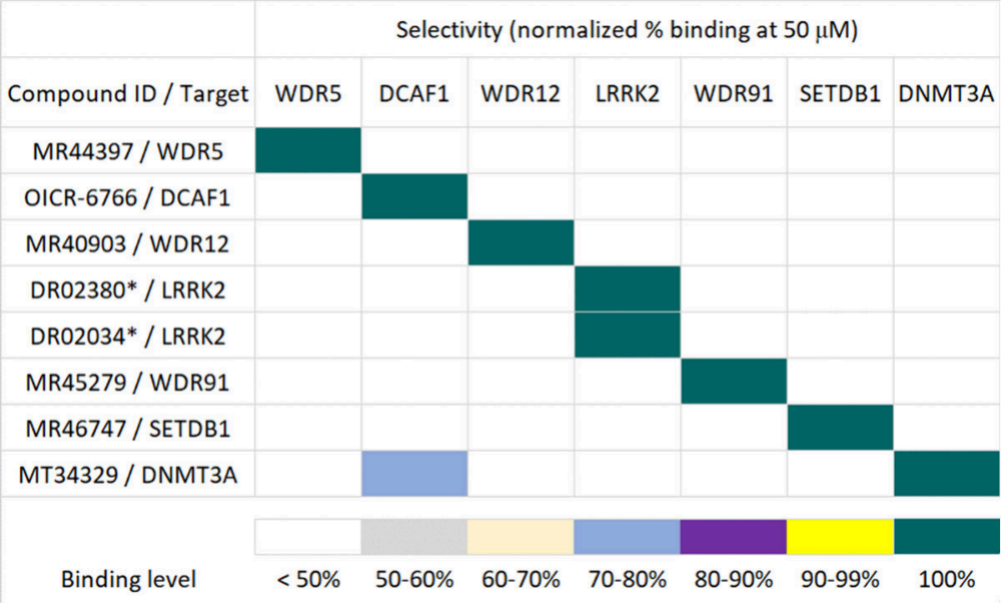
Selectivity Profiles of Discovered Hits^a^

a In the leftmost column, each row is named for the discovered hits and their corresponding targets. Each column under the heading “Selectivity” shows the normalized percent binding as determined by SPR at a compound concentration of 50 μM. A dose-response experiment was conducted if off-target binding was > 50% (Figure S8). *Visible precipitation in DMSO stock solution which redissolved with gentle heating. DLS (performed in 20 mM HEPES pH 7.4, 150 mM NaCl, 3 mM EDTA) indicates that DR02380 should be soluble and have no aggregation up to 200 μM. However, DLS shows that solubility and aggregation of DR02034 is compromised at 200 μM.

With cell-free characterization of compound affinity, binding mode, and a measure of selectivity in hand, we describe cellular assays to evaluate putative PPI antagonists.

### Cellular Assays to Test Compound Activity

**Figure 7:**
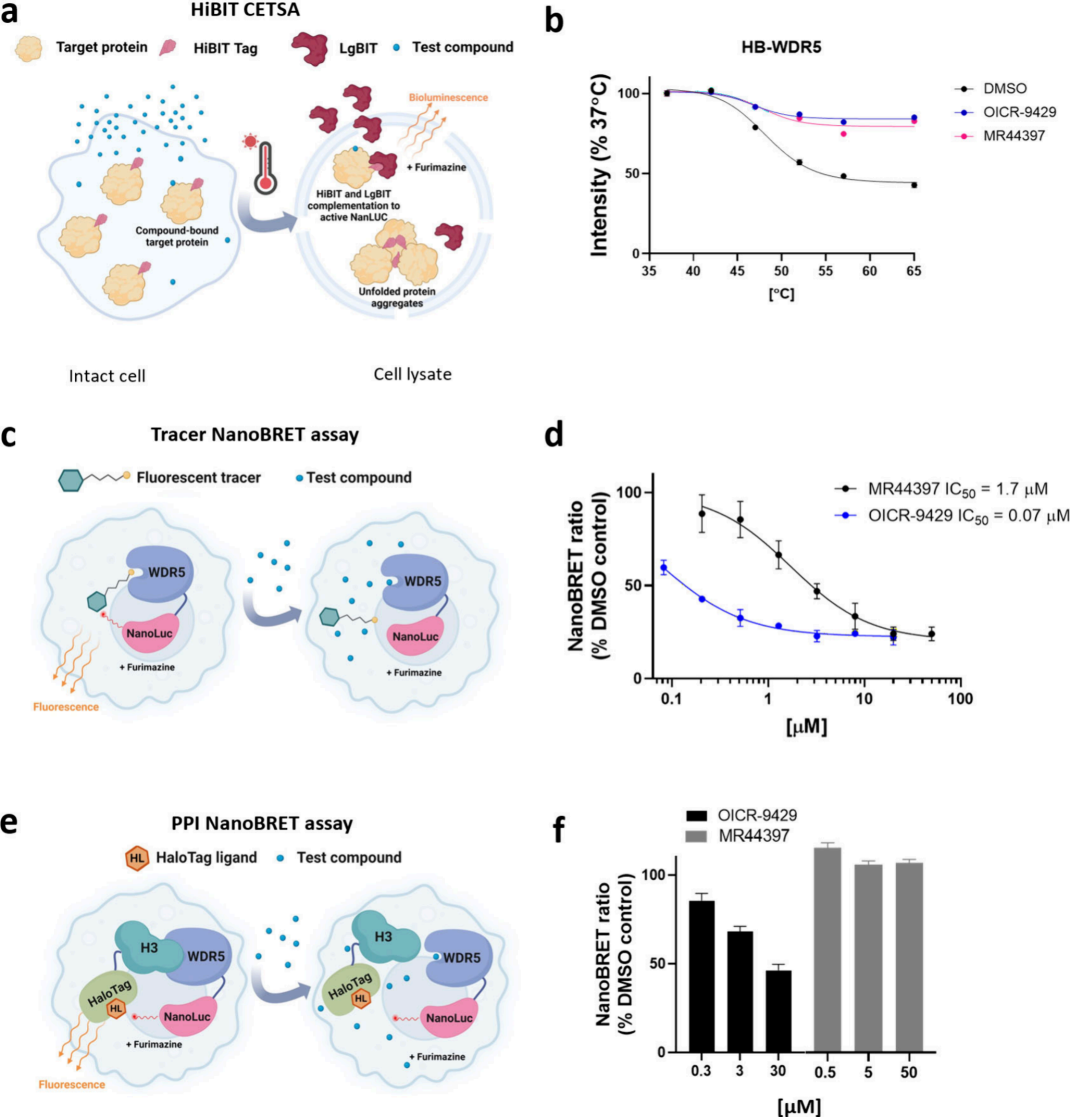
MR44397 cellular WDR5 target engagement. (**a**) Schematic representation of CETSA.^49^ (**b**) MR44397 and the WDR5 chemical probe OICR-9429 (positive control) stabilize WDR5 in HEK293T cells. Cells were transfected with N-terminally HiBiT (HB) tagged WDR5 and incubated with 50 μM MR44397 and 20 μM OICR-9429 for 1 h. After heating for 3 min in an indicated temperature gradient, cells were lysed and incubated with LgBIT. The results are MEAN± SD of NanoLuc bioluminescence signal (*n* = 4). (**c**) Schematic representation of fluorescent tracer nanoBRET assay.^48^ (**d**) Dose–response of OICR-9429 and MR44397 competition with a fluorescent tracer compound indicated by decreased nanoBRET ratio of WDR5 and tracer interaction^65^ in HEK293T cells. Cells were transfected with N-terminally NL-tagged WDR5 for 24 h and incubated with 1 μM fluorescent tracer and indicated compound concentrations for 2 h MEAN± SD (*n* = 4). (**e**) Schematic representation of a nanoBRET-based PPI assay. (**f**) In contrast to WDR5 chemical probe OICR-9429 (positive control), MR44397 does not decrease the nanoBRET ratio between WDR5 and histone H3 in HEK293T cells. Cells were cotransfected with C-terminally NanoLuc (NL) tagged WDR5 and C-terminally HaloTag (HT) tagged histone H3 for 24 h and incubated with indicated compound concentrations for 4 h. The results are MEAN± SD (*n* = 4).

An important step in the drug discovery process is confirming the target engagement of a small molecule in a native cellular environment. To develop specific cellular assays for individual WDR proteins we utilized the NanoLuc Luciferase (NL) technology, including HiBiT Cellular Thermal Shift Assay (HiBiT CETSA), NanoBRET-based tracer assays, as well as PPI NanoBRET assays.^61^ Below we describe the suite of cell assays developed for WDRs and provide examples demonstrating target engagement by WDR5 ligands (Figure 7).

To measure the effect of small molecule binding on protein thermal stability, we used a split NanoLuc reporter system (HiBiT CETSA).^62,63^ In this assay, the target protein, which is fused to the 11 amino acid HiBiT tag, is overexpressed in cells which are then subjected to a thermal challenge followed by complementation with LgBIT comprising the remainder of the NanoLuc enzyme, thus allowing the level of soluble protein to be quantified from the resulting luminescence signal (Figure 7a). As shown in Figure 7b, the newly discovered ligand MR44397 and the chemical probe OICR-9429 thermally stabilized exogenously expressed HiBiT-WDR5 in HEK293T cells, indicating cellular permeability and binding to WDR5.

To assess the suitability of this assay for the wider collection of WDR proteins we first compared our systematic DSF data on WDR protein melting temperatures (*T*_m_) (Table S5) and the *T*_m_s for several HiBiT-tagged WDR proteins transfected in HEK293T cells (Table S12) relative to those predicted in the Meltome database.^64^ This comparison indicates a reasonable agreement between endogenous protein *T*_m_ and transfected HiBit-tagged WDR protein *T*_m_. For example, RBBP5 has a high (∼60 °C) *T*_m_ in the Meltome, HiBit CETSA, and DSF (Tables S5, S12). Since temperatures above 60–65 °C can impact cellular permeability, the method is not suitable for proteins with high melting temperatures. RBBP4, RBBP5, WDR41, or WDR82 are examples of such WDR proteins (Tables S5, S12). In other cases, compound binding did not result in protein stabilization of the full-length protein, making the HiBiT CETSA assay not suitable for cellular target engagement. This problem may be resolved by the utilization of targeted domains instead of full-length proteins. For example, OICR-8268 potently stabilized the DCAF1-WD40 domain in DSF and HiBiT CETSA assays but not full-length DCAF1.^50^

Another way to measure target engagement in cells is a tracer-based NanoBRET assay using bioluminescence resonance energy transfer (BRET) from NL to a fluorescent tracer molecule.^66^ In this assay, the endogenously tagged or exogenously expressed NL-tagged protein of interest acts as an energy donor in the presence of the furimazine substrate, and a cell-permeable small molecule ligand of the target protein covalently conjugated to the fluorescent dye (tracer) acts as an energy acceptor (Figure 7c). The competition of the test compound with the tracer for binding to the protein of interest results in a decrease in NanoBRET signal between the protein and a tracer and allows the determination of cellular permeability, apparent binding affinity, and residence time of the test compound in intact as well as permeabilized cells.^66^ As an example, the IC_50_ determination in the tracer NanoBRET assay is demonstrated using the WDR5 tracer (Figure 7d).^65^ Both MR44397 and the chemical probe OICR-9429 decreased the NanoBRET ratio in a concentration-dependent manner with different potencies. This assay confirmed cellular permeability and target engagement of MR44397 with an IC_50_ of 1.7 μM.

In cases where the WDR protein has a known interaction partner that is expected to compete with the ligand of interest, cellular activity and target engagement can be tested with a PPI NanoBRET assay.^67,68^ In the PPI NanoBRET assay, one of the proteins of interest is tagged with NL (energy donor), and the other protein of interest is tagged with HaloTag (HT) that binds covalently to a fluorescent chloroalkane ligand and acts as the BRET acceptor if the two proteins are in close proximity (Figure 7e). Besides the measurement of the interactions between full-length proteins, the assay can be modified to measure the BRET ratio between short peptides or isolated domains.^48^ An advantage of using individual domains is that the NL and HT tags are likely to be closer upon protein interaction with a resultant higher BRET signal compared with larger full-length proteins where the tags may be more distal to the interaction site.

Figure 7f shows the PPI NanoBRET assay for the WDR5-Histone H3 interaction competed with MR44397, and the chemical probe OICR-9429. Although both compounds bind to the central pocket of WDR5 and displace the H3 peptide in vitro (Figure 2), only the OICR-9429, which is 20 times more potent than MR44397 in the tracer NanoBRET assay (Figure 7d) was able to decrease the NanoBRET ratio. This result exemplifies our observations that, in general, potent (submicromolar) compounds are required to observe robust disruption of a PPI.

We have developed several additional PPI NanoBRET assays for WDRs which will be useful as potent ligands are developed in the future (Table S13). If possible, to further validate the assay and determine the assay window, we also utilized genetic mutations in donor or acceptor proteins known or predicted to disrupt the interaction. For example, a previously published R29D mutation within CORO1A^69^ resulted in a decrease in NanoBRET ratio with ACTB by 50% and a double mutation predicted from a cocrystal structure of FBXW7 (R465E, R505E) completely abolished the NanoBRET signal with Cyclin E1 (Table S13).^70^

## Conclusions

In the field of drug discovery and development of chemical biology tools, the initial hit-finding phase can often be long, tedious, and particularly prone to experimental artifacts. Although all assays suffer to some extent from these issues, a robust and generalizable hit-finding strategy, starting with high-quality protein and that can be executed at scale, will be highly enabling for many applications in medicine and chemical biology. We applied a DEL-ML hit-finding strategy to 16 WDRs with a range of druggability^35^ scores (Figures 1b–d), and two non-WDRs with ligandable PPI domains as reference points.

To our knowledge, this is the first time that a large cohort of proteins from a single protein class was subjected to hit-finding (by DEL-ML) in a systematic and coordinated manner. Computational druggability analysis using AlphaFold models of over 300 human WDR proteins suggested that ∼50% of WDRs are indeed ligandable. This is likely a conservative estimate considering that cryptic pockets are often hidden in apo WDR structures^31^ (Figure S2, Tables S3, S6, S8). It is worth noting that coverage of chemical space, as well as the hit-finding approach, will invariably influence whether small molecule ligands are discovered for WDRs (or any member of any protein family).

We did not observe a trend between the theoretical and experimental druggability of the central pocket. From the druggability score (Figure 1d, Table S8), we expected central pocket ligands to have been discovered for all WDRs with DLID > 0. If we consider the targets in Figure S2, WDR5, EED and CDC20 have DLID values > 0, while BTRC has a DLID value < 0. Nevertheless, there are public domain ligands that bind near to the central pocket for WDR5, EED and BTRC but none are reported for CDC20. For the latter, the only public domain ligand (apcin) binds in a side pocket (Figure S2d). We report novel, selective hits for previously unliganded WDRs, of which DCAF1 and LRRK2 are predicted to be below the “ligandable threshold” (Figure 1d, Table S8). More surprisingly, the DCAF1 ligand binds deep in the central pocket (PDB ID: 7UFV). Additionally, we discovered central pocket ligands for WDR12 and WDR5 (both with DLID scores > 0), but only side pocket ligands for WDR91 which is predicted to have the most druggable central pocket of the 16 WDRs (Figure 1d, Table S8). Our discovery of ligands for almost half of the tested WDR domains clearly demonstrates their druggability and the promise of the DEL-ML approach for this family.

With the exception of WDR5 we did not systematically explore whether our new ligands could modulate native protein function in cell-free systems (such as displacement assays) or in cell-based assays because our primary focus was primarily on assessing ligandability and hit-finding. However, it is quite likely that potent ligands in the central pocket of the WDR would modulate key PPI functions. Moreover, even ligands that are not able to modulate a PPI are nevertheless highly valuable starting points for developing proximity-based pharmacological agents, such as PROTACs and molecular glues. Notably, many WDRs function as substrate binding modules of E3 ligases to recruit substrates for polyubiquitylation and degradation by the proteasome.^3^ This includes most substrate binding domains of the Cullin 4 E3 ligase family,^16,71^ the FBXW class of Cullin 1 and 7 E3 ligase families such as FBXW5, FBXW7, and some non-Cullin E3 ligases such as RFWD2 and RFWD3. Additionally, several other WDR proteins such as WDR5, EED and WDR12^17^ interact with DDB1, which is itself a WDR domain-containing substrate adaptor of the CUL4 E3 ligases. Therefore, new small molecule ligands that bind to WDR domain-containing E3 ligases may expand the repertoire of E3 ligases available for PROTAC development.^72^

## Significance

This work was inspired by the Target 2035 initiative to discover pharmacological modulators for the human proteome.^20,23^ This “dream” was expressed 14 years ago by Crews who called for new chemical biology methodologies to target the “undruggable proteome” with small, drug-like molecules to enable proximity-mediated drug discovery.^73^ Much progress has been made since 2010, including the development of DEL-ML and other computationally supported methods. However, there is still a long way to go.^74^ Here we provide a roadmap for the rapid and systematic assessment of ligandability across protein families. This target-focused approach requires the large-scale production of high-quality protein, crystallography, hit-finding, and orthogonal confirmation of binding. Indeed, we have purified 95 WDRs at scale with at least one affinity tag, solved 21 3D structures, discovered at least one hit for each of seven targets, and developed 24 cellular target engagement assays. The ensemble of resources presented here will further enable exploration of the unliganded WDR protein family. More importantly, it is a blueprint for target-focused ligand discovery for the wider human proteome.

## Experimental Section

### Informatics and Druggability

Using InterPro IDs: IPR001680 IPR036322 IPR015943 as the search query, a list of 349 WDR domain proteins and the number of publications and the number of articles associating each protein with specific disease areas were sourced from chembioport.thesgc.org, which extracts data from the NCBI’s gene2pubmed database (Table S2).^75^ The amino acid sequence of each WDR domain protein was sourced from UniProt and a multiple sequence alignment (MSA) was generated using ICM-Pro 3.9–3a (Molsoft, San Diego). The data collected and MSA were then used to generate annotated phylogenetic trees using iTOL v6.

AlphaFold V2.0 models for each of the 349 WDR proteins (Table S3) were downloaded from https://alphafold.ebi.ac.uk/.^33,76^ Structures were unavailable for eight proteins (UniProt IDs: P50851, Q5T4S7, Q6ZNJ1, Q6ZS81, Q8IZQ1, Q8NFP9, Q9H799, and Q99698) and thus were excluded from the analysis. After visual inspection, six proteins were excluded from further analysis due to the lack of a complete “donut”-shaped domain (UniProt IDs: P18583, P55735, O75460, Q76MJ5, Q9NZJ5, and Q96EE3). When multiple WDR domains were found in a protein, they were all analyzed. Each WDR domain was subjected to the ICMPocketFinder algorithm using ICM-Pro 3.9–3a (Molsoft, San Diego) to predict the location of potential pockets and to obtain the drug-like density (DLID) score.^35^ The domain was then superimposed with a canonical WDR structure (WDR5, PDB ID: 4QL1) with 4 dummy atoms placed down the middle of the central pocket. The DLID value of the pocket with the smallest distance to any of these 4 atoms was reported as the druggability index for the central pocket. For proteins with multiple WDR domains, the highest DLID score was reported. Proper superimposition was confirmed through visual inspection. To assess the predictive confidence of the pocket, the number of residues within 5Å from the pocket with an AlphaFold confidence score (pLDDT) < 70 were reported. DLID scores for pockets where no such residues are found are expected to be more reliable.

### Biotechnology

Individual expression clones are detailed in Table S3 and are available from Addgene. Protein expression and purification summaries are detailed in Table S4 and deposited in Zenodo.^31^

### Structural Biology

Twenty-one high resolution structures have been deposited in the PDB (Table S6). The data collection and refinement statistics are given in Table S7.

### DNA Encoded Chemical Library Selection

Affinity mediated selections were made from pools ranging from 19 to 65 DNA-encoded libraries.^77^ For each His6-tagged target, the DEL deck was combined with the protein, and incubation occurred in solution prior to capture on the IMAC matrix; for biotinylated targets, the target was captured on the streptavidin matrix prior to blocking with biotin and the subsequent introduction of the DEL deck (Table S9). For each target an additional selection condition containing no target was performed in parallel to identify matrix binders. For some targets an additional selection condition containing target protein with additive, e.g., a competitor, was performed in parallel (Table S9).

For each IMAC selection (no target, target, or target with competitor), purified protein was preincubated for 30 min with or without additive with the DEL deck (40 μM) for 1 h in a volume of 60 μL in 1× selection buffer, and then a separate ME200 tip (Phynexus) containing appropriate affinity matrix was prewashed three times in 200 μL of fresh 1× selection buffer. Each selection was separately captured with 20 passages over the ME200 tip over 0.5 h. The bound protein and associated library members were captured on the ME200 tip and washed eight times with 200 μL of fresh 1× selection buffer. Bound library members were eluted by incubating the ME200 tip with 60 μL of 1× fresh selection buffer at 85 °C for 5 min. The elution was incubated with 20 passages over a fresh, prewashed ME200 tip containing the appropriate affinity matrix to remove any eluted protein. The selection process was run for a second time using the eluate of the first selection in place of the input DNA-encoded library and using an appropriate fresh target or no target.

For streptavidin matrix selections (no target, target, or target with competitor), purified protein was preincubated for 30 min with or without additive in a volume of 60 μL in 1× selection buffer. A separate ME200 tip (Phynexus) containing the affinity matrix was prewashed three times in 200 μL of fresh 1× selection buffer. Proteins were captured with 20 passages over the ME200 tip for a total of 0.5 h. The bound protein captured on the ME200 tip was washed two times with 200 μL of fresh 1× selection buffer (with 50 μM biotin), and then the DEL deck (40 μM) was introduced in a volume of 60 μL in 1× selection buffer with or without additive was passaged over the ME200tip 40 times over the period of an hour. ME200 tips were then washed eight times with 200 μL of fresh 1× selection buffer. Bound library members were then eluted by incubation with 60 μL of 1× fresh selection buffer at 85 °C for 5 min. The elution was then subjected to 20 passages over a fresh, prewashed ME200 tip containing an appropriate affinity matrix to remove any eluted protein. The selection process was run for a second time using the eluate of the first selection round in place of the input DNA-encoded library and using appropriate fresh target or no target. 1x Selection Buffer, also referred to as Cytoplasmic Selection Buffer (CSB), is comprised of 20 mM HEPES, 134 mM KOAc, 8 mM NaOAc, 4 mM NaCl, 0.8 mM Mg(OAc)_2_, 0.02% Tween-20, 1 mgmL^–1^ Sheared Salmon Sperm DNA, 5 mM Imidazole, 1 mM TCEP, pH 7.2.

For both IMAC and Streptavidin matrix selections, the eluate of the second round of selection was PCR amplified and sequenced.^77^ Sequence data were parsed, error-containing sequences were disregarded, amplification duplicates were removed, and building block and chemical scheme encodings were decoded and reported along with associated calculated statistical parameters.

The immobilization efficiency of proteins during selection experiments was determined by SDS-PAGE. Input sample is identical with the initial solution containing protein in 60 μL of 1× selection buffer prior to capture with 20 passages over the ME200 tip. Flow-through sample is the solution containing protein in 60 μL of 1× selection buffer after capture with 20 passages over the ME200 tip from selection. Resin sample is the resin removed from the ME200 tip and mixed to resuspension in 60 μL of 1× selection buffer. All proteins demonstrated ≥ 80% purity and ≥50% fraction of input protein immobilized (Figure S9).

#### ML Models Generated Using Random Forest

For the random forest models (Table S10), disynthons were classified into two groups: “non-hits” (label = 0) and either “hits” or “competitive hits” (label = 1), depending on whether the selection experiment included a condition with a competitive binder. Thirty models were trained with one million negative examples randomly sampled from the “non-hits” and 90% randomly sampled from the “hits” or “competitive hits” as positive examples to make up the training set for each model. 10% of this total set of examples was held out as a test set and was not seen during training. The positive (label = 1) examples were oversampled by including them twice during the training for each model. Each molecule in the training and test sets was featurized using the Morgan fingerprint algorithm as implemented in the RDKit cheminformatics package with radius = 2 and bit count = 1024.^78^ The models were trained using the RandomForestClassifier class in the scikit-learn Python package.^79^ Nondefault hyperparameters for the models included n_estimators = 2000 and min_samples_split = 5. Of the thirty trained models, the model with the highest accuracy on the test set was chosen for inference on commercial catalogs.

#### ML Models Generated Using GCNN

The GCNN model in this study (Table S10) is based on that in McCloskey et al.,^24^ with several improvements referred to as GCNN V1, V2 and V2.1. We elaborate on all 3 versions below.

#### Label Assignment for ML

DNA Encoded Libraries molecules were aggregated into disynthons and enrichment values were calculated following the approach detailed in McCloskey et al.^20^ Subsequently, each disynthon was assigned to one of five distinct classes: competitive hit, noncompetitive hit, promiscuous binder, matrix binder, or nonhit.^20^ For targets where a competitor molecule was incubated during a DEL affinity screen, the positive training examples for machine learning were designated as the “competitive hit” class. For the targets without competitor molecules, a target hit class replaced both competitive hit and noncompetitive hit classes, resulting in four instead of five classes.

**GCNN V1** adopted the model from McCloskey et al.^24^ with the following updates: (1) The model training was migrated from CPU to tensor processing units (TPU) to take advantage of TPU’s strength in synchronous training. (2) In addition to ensembling the cross-validation fold models as described in McCloskey et al.,^24^ we implemented two extra levels of model ensembling to improve overall reliability: (a) We used TPU-supported graph partitioning for manual model parallelism, training 8 models with independently randomized weight initialization on individual TPU cores. The ensembled prediction is the median of prediction scores from these 8 models. (b) We further replicated the full training process 3 times, and the final prediction score is the median of predictions from the 3 runs, each with 8 TPU model replicas, and the cross-validation fold models.

**GCNN V2** built upon GCNN V1 with the following key improvements: (1) Instead of using the maximum ROC-AUC we utilized the top_100_actives metric. This metric counts the number of active examples within the top 100 ranked predictions for the hit class. After training models for each cross-validation fold, we selected the checkpoint weights with the highest top_100_actives score on the tuning set. (2) Along with changing the model selection metric, we also updated training hyperparameters: (a) Learning rate was increased from 0.0006 to 0.1. (b) Training steps were increased from 30,000 to 1,000,000, with checkpoints saved every 100,000 steps instead of every 3,000 steps.

**GCNN V2.1** is an ensemble model combining the predictions from GCNN V2 and a molecular-fingerprint-based deep neural network (DNN). The fingerprint DNN architecture consists of 2 fully connected layers of sizes (2000, 100) with rectified linear activation function (ReLU) and the input features are 2048-bit Morgan Fingerprint with radius of 3 (ECFP6_2048). The ensemble procedure involves first retrieving the top 100,000 compounds using the GCNN V2 model predictions, and then filtering out 50% of these compounds based on the lower DNN prediction scores, and from the remaining 50,000 compounds, following the same protocol of property filtering and diversity selection, ranked by GCNN V2 predictions, to select the compounds for purchase and testing.

#### ML Models Generated Using ArtemisAI

Where the model type is specified as “ArtemisIAI” (Table S10), the DEL selection data were featurized in a range of different formats, and each format was then used to build a collection of models. These models were evaluated and, based on performance, included in a model ensemble used to score commercial catalogs compounds. A diverse set of the highest scoring compounds was ordered and assayed.

#### DEL-ML-Driven Hit Expansion

For promising hit compounds like those found for WDR12, DCAF1, and WDR91, we performed automated hit expansion using the GCNN model to search for and prioritize analogs of the initial hit. The search was performed in the large Enamine REAL library of 1.9 billion commercially available compounds, avoiding expensive bespoke synthesis. First, we identified analogs from the Enamine REAL library with ECFP6 Tanimoto similarity above a threshold to the original hit. We then used the GCNN prediction score to rank these compounds. To balance exploiting structural similarity and exploring diversity, we also applied DISE selection with a 0.2 radius cutoff to increase analog diversity.

### Computationally Based Hit Expansion

The EnamineReal commercially available structural space most similar to the 4 initial DCAF1 hits (Tanimoto Score > 0.35 using ECFP6, ∼87 thousand compounds) was docked (ICM and OpenEye ORION platform) in the X-Ray structure and compounds with high docking scores were ordered and assayed. A similarity set was also selected from the highest docking scores (ICM and OpenEye ORION platform) from a 10 M random sampling of EnamineReal. Lastly, a 3D shape-based ROCS hit expansion search (Openeye ORION platform) of the 4 original hits was conducted and a set of compounds with good shape complementarity (Tanimoto combo score > 1.4) to the original hits was ordered and assayed.

### Compound Purity

All reported compounds have a purity of at least 95% at 254 nm (Figure S10). The purity of the compounds was determined by using a liquid chromatograph equipped with a diode array and a mass spectrometer detector (Waters Acquity SQD mass spectrometer).

### Surface Plasmon Resonance (SPR)

The binding affinity of compounds was assessed by SPR (Biacore T200 or Biacore 8K, Cytiva Inc.) as previously described.^39^ SPR running conditions (Table S5) vary slightly, depending on the specific compounds and protein targets.

### Differential Scanning Fluorimetry (DSF)

WDR5 was diluted to 0.1 mg/mL in buffer (100 mM HEPES, 100 mM NaCl, pH 7.5) in the presence of 5 × SYPRO Orange dye (Life Technologies, S-6650) and serially titrated MR44397 (up to 200 μM) in a total volume of 20 μL in a white polypropylene 384-well plate (Axygen, #UC500). DSF was performed in a Light Cycler 480 II (Roche Applied Science, Penzberg, Germany) using a 4 °C/min temperature gradient from 20 to 95 °C. Data points were collected at 1 °C intervals. DSF data was fitted to a Boltzmann sigmoid function and *T*_m_ values were determined as previously described.^6^

### Fluorescence Polarization (FP) Assay

WDR5 (H3 1–15 FP: 300 nM, WIN and RBBP5 FP: 5 μM) was incubated with 40 nM of fluorescent FITC labeled peptide-H3 1–15, -WIN (GSARAEVHLRKS) or - RBBP5 (371–380 aa, EDEEVDVTSV) in the presence or absence of serial titration of MR44397 (up to 100 μM) for 30 min at ambient temperature, and fluorescence polarization was then measured and analyzed as previously described.^80^

SETDB1 TTD (197–403) 5 μM was incubated with 40 nM of 5′ FITC- H3K9me2K14ac (1–25) with serially diluted MR46747 compound at 200 μM top concentration (12 points 3-fold dilution). Final DMSO in the reaction mixture was maintained at 2%. Following a brief spin down and incubation for 30 min at room temperature fluorescence polarization ratio measurements were obtained as reported previously.^80^

### Biolayer Interferometry (BLI) Assay

The BLI assay was used to evaluate the binding kinetics of MR44397 against WDR5. All assays were run on the same Octet Red96 instrument at 25 °C while being shaken at 1000 rpm. BLI assay buffer consists of 0.1% BSA in 10 mM HEPES, pH 7.4, 150 mM NaCl, 0.05% Tween 20, 3 mM EDTA. Antibiotin super streptavidin (SSA) biosensors (part no: 18–5057, FORTÉBIO, Fremont, California, United States) were loaded into the columns of a biosensor holding plate and prehydrated in BLI assay buffer for 10 min. Black 96-well microplates were loaded with 200 μL per well. Following a 60-s equilibration step with antibiotin super streptavidin (SSA) biosensors in buffer, biotinylated WDR5 was loaded for 300 s. The SSA were then moved into wells in the following sequence: (i) a baseline step with fresh assay buffer for 60 s, (ii) MR44397 for a 60 s association step, and (iii) assay buffer for a 120 s dissociation step. Binding curves from the association and dissociation steps were fitted using a 1:1 binding model to determine *K*_D_ values using the Octet Data Acquisition software.

### Dynamic Light Scattering (DLS)

The solubility of compounds was estimated by DLS that directly measures compound aggregates and laser power in solution. Compounds were serially diluted directly from DMSO stocks, then diluted 50x into filtered HBS-E (20 mM HEPES pH 7.4, 150 mM NaCl, 3 mM EDTA) for analysis by DLS (DynaPro DLS Plate Reader III, Wyatt Technology, USA) as previously described.^81^ The solubility is listed alongside binding data in Table S10.

### Nuclear Magnetic Resonance (NMR) Spectroscopy

All spectra were collected at 298K on a Bruker AvanceIII spectrometer, operating at 600 MHz, and equipped with a QCI probe. The binding of fluorinated compounds was assayed by looking for the broadening and/or perturbation of ^19^F resonances in 1D spectra upon addition of LRKK2 (at protein to compound ratios of 0.5:1 to 4:1) in PBS buffer (pH7.4, 137 mM NaCl, 2.7 mM KCl, 10 mM Na_2_HPO_4_, 1.8 mM KH_2_PO_4_, and with 5% D_2_O). Two to four thousand transients were collected with an acquisition period of 0.2 s, over a sweep width of 150 ppm, a relaxation delay of 1.5 s, and using 90° pulses centered at −120 ppm. The concentrations of the compounds in both reference and protein-compound mixtures were 5–10 μM. TFA (20 μM) was added as an internal standard for referencing. Prior to Fourier transformation, an exponential window function was applied (lb = 1 to 3) to the FID. ^1^H-^15^N HSQC spectra of ^15^N-labeled DNMT3A-PWWP were acquired to confirm the interaction of compounds by looking for the perturbation of amide resonances. The concentration of the labeled construct was 100 μM, and buffered in 50 mM Tris pH 7.5, 250 mM NaCl, 5% Glycerol, and 1 mM DTT containing 2.5% DMSO; compounds were at 500 μM. 128 indirect FIDs were acquired with 96 transients collected over a 32 ppm ^15^N sweep width. Squared cosine window functions were applied in both dimensions prior to the Fourier transformation. Spectral processing was performed at the workstation using the software Topspin3.5.

### Hydrogen–Deuterium Exchange Mass Spectrometry

HDX-MS was performed as previously described using the PAL3 Autosampler (CTC Analytics AG) coupled to an M-Class UPLC (Waters Corp., U.K.) and SELECT SERIES Cyclic IMS Mass Spectrometer (Waters Corp., U.K.) in HDMSe mode.^82^ Briefly, samples (5 μM WDR12 or 5 μM WDR12 + 100 μM MR44915) were labeled (10 mM Phosphate Buffer pD 7.4, 150 mM NaCl) at 20 °C for 0, 1, 10, 30, and 60 min. The HDX reaction was then quenched (100 mM Phosphate Buffer, pH 2.5) at 0 °C for 1 min prior to proteolysis (Enzymate BEH Pepsin column, Waters Corp., U.K.), desalting (ACQUITY UPLC BEH C18 VanGuard Precolumn, Waters Corp., U.K.), and reverse-phase separation (ACQUITY UPLC BEH C18 Column, Waters Corp., U.K.). Data processing was conducted using ProteinLynx Global Server 3.0.3. and DynamX 3.0 (Waters Corp., U.K.), and visualization was done using PyMol 2.5.0 (Schrodinger, LLC). For a cumulative ΔHDX-MS peptide signal to be considered statistically significant, it must have exceeded the cumulative propagated error (2 σ, *n* ≥ 2).

### NanoBRET-based Protein–Protein Interaction (PPI)

A detailed protocol was recently published.^48^ Briefly, HEK293T cells were seeded in 96-well white plates (4 × 10^4^ cells/well, Corning) and cotransfected with C-terminally NL-tagged WDR5 (0.001 μg/well) and C-terminally HT-tagged histone H3 (0.1 μg/well) or N-terminally NL-tagged WDR5 (0.001 μg/well), C-terminally HT-tagged RBBP5 (0.03 μg/well), and empty vector (0.07 μg/well) using XtremeGene HP transfection reagent, following manufacturers’ instructions. The next day the medium was replaced with DMEM (no phenol red) supplemented with 4% FBS, 100 U/mL penicillin, 100 μg/mL streptomycin, ±1000-fold diluted HaloTag NanoBRET 618 Ligand (Promega) and incubated with indicated compound concentrations or DMSO control for 4 h. Next, 100-fold diluted NanoBRET Nano-Glo Substrate (Promega) was added, and donor emission at 450 nm and acceptor emission at 618 nm were read within 10 min of substrate addition using a ClarioStar plate reader (Mandel Scientific). The NanoBRET ratio was determined by subtracting 618/460 (acceptor/donor) signal from cells without NanoBRET 618 HaloTag Ligand x 1000 from 618/460 signal from cells with NanoBRET 618 Ligand x 1000.

### Cellular Interaction between Small Molecules and Proteins

#### NanoLuciferase-Based Cellular Thermal Shift Assay

A protocol was recently published.^49^ Briefly, HEK293T cells were seeded in 6-well white plates (8 × 10^5^ cells/well, Corning) and transfected with N-terminally HiBiT-tagged WDR5 (0.2 μg/well) and empty vector (1.8 μg/well) using XtremeGene HP transfection reagent, following manufacturers’ instructions. The next day cells were trypsinized and resuspended in optiMEM (no phenol red, Gibco) at the density of 2 × 10^5^ cells/mL. After compound addition (50 μM of MR44397 and 20 μM of OICR-9429) or DMSO, the cells were aliquoted in a 96-well PCR plate (one well per intended temperature, 50 μL), covered with breathable film and incubated for 1 h at 37 °C 5% CO_2_. Next, cells were heated in the VerityPro thermocycler (Thermo Fisher Scientific) as follows: 1 min at 22 °C and 3 min at the indicated temperature gradient, followed by a cooling step to chill samples to RT (22 °C). Next, 50 μL of lytic buffer was added (2% NP-40, protease inhibitors (Roche), 0.2 μM LargeBiT (produced in house) in optiMEM (no phenol red)). After 10 min incubation at RT, 25 μL/well of 100-fold diluted NanoBRET Nano-Glo Substrate (Promega) was added and transferred to 384-well white plates in quadruplicates (20 μL/well). The luminescence signal was read in a ClarioStar plate reader (Mandel Scientific). Data were fitted to obtain apparent *T*_agg_ values using the nonlinear curve fit (e.g., Boltzmann Sigmoid equation) using GraphPad Prism software.

#### Tracer-Based NanoBRET Assay

HEK293T cells were plated in a 6-well plate (8 × 10^5^ cells/well) and transfected with 0.2 μg of N-terminally NL-tagged WDR5 vector and 1.8 μg of empty vector using XtremeGene HP transfection reagent, following manufacturer’s instructions. The next day cells were trypsinized and resuspended in optiMEM (no phenol red) at 2 × 10^5^ cells/mL density with 1 μM of WDR5 tracer.^65^ Compound serial dilutions were prepared in DMSO and added to the cells. Cells were transferred to 384-well low-binding white plates (20 μL/well, Corning) in quadruplicates. After 2 h, 5 μL/well NanoBRET Nano-Glo Substrate (Promega) and Extracellular NanoLuc Inhibitor (Promega) diluted in optiMEM (no phenol red) 100-fold and 300-fold, respectively, was added. The donor emission at 450 nm and acceptor emission at 618 nm were read immediately after substrate addition and shaking of the plate for 20 s using a ClarioStar plate reader (Mandel Scientific). NanoBRET ratios were calculated by subtracting the mean of the 610 nm/460 nm signal from cells without tracer × 1000 from the 610 nm/460 nm signal from cells with tracer × 1000. Data were fitted to obtain IC_50_ values using the nonlinear curve fit using GraphPad Prism software.

### Experimental Procedure of 1-Acetyl-*N*-((2-(2-chlorophenoxy)pyridin-4-yl)methyl)indoline-5-sulfonamide MR44915

#### Reaction Scheme

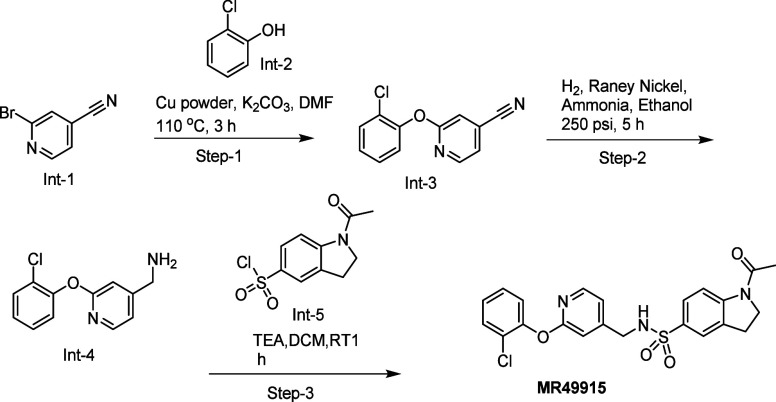



#### Step-1: Synthesis of 2-(2-Chlorophenoxy) isonicotinonitrile (Int-3)

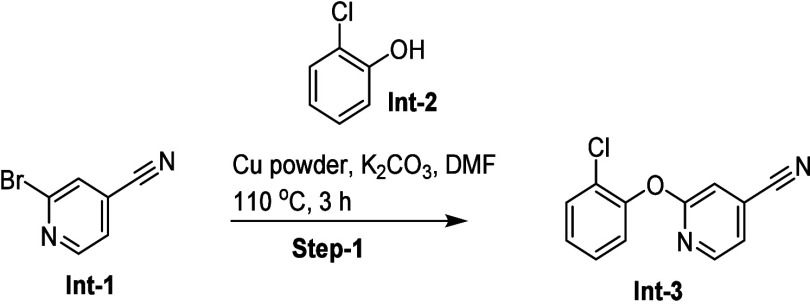



To a mixture of 2-bromoisonicotinonitrile **(Int-1)** (0.2 g, 1.09 mmol, 1.0 equiv) and 2-chlorophenol **(Int-2)** (0.2 g, 1.63 mmol, 1.5 equiv) in *N, N*-dimethylformamide (2 mL), were added potassium carbonate (0.23 g, 1.63 mmol, 1.5 equiv) and copper powder (0.04 g, 0.655 mmol, 0.2 equiv), and the mixture was stirred for 3 h at 110 °C. The reaction mixture was poured into water and extracted with ethyl acetate dried over sodium sulfate and concentrated under reduced pressure to give the crude compound, which was purified by column chromatography (SiO_2_, ethyl acetate:hexanes = 1:10 to 2:10) to give 2-(2-chlorophenoxy) isonicotinonitrile **(Int-3)** (0.17 g, yield: 67.63%) as a brown solid.

**LC-MS**: *m*/*z* = 232.8 [M + 2H]^2+^.

#### Step-2: Synthesis of (2-(2-Chlorophenoxy) pyridin-4-yl) methanamine (Int-4)


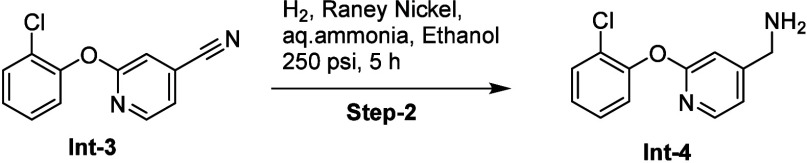
To a solution of 2-(2-chlorophenoxy) isonicotinonitrile (**Int-3**) (0.17 g, 0.737 mmol, 1.0 equiv) in ethanol (11.2 mL) in an autoclave under a nitrogen atmosphere were added aqueous ammonia (1.1 mL) and Raney nickel (0.2 g) and the mixture stirred under 250 psi pressure of hydrogen for 5 h. The reaction mixture was filtered through a Celite pad, and the filtrate was concentrated under reduced pressure to give crude product (2-(2-chlorophenoxy) pyridin-4-yl) methanamine **(Int-4)** (0.16 g, yield: 92.50%) as a brown solid, which was used in the next step without further purification.

**LC-MS**: *m*/*z* = 235.0 [M + H]^+^.

#### Step-3: Synthesis of 1-Acetyl-*N*-((2-(2-chlorophenoxy)pyridin-4-yl)methyl)indoline-5-sulfonamide MR44915

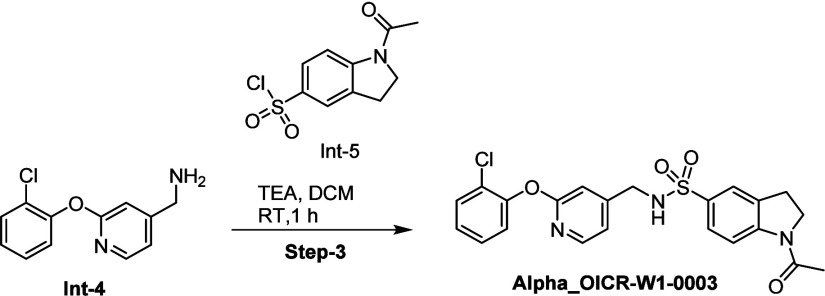



To a cooled to solution (0 °C) of (2-(2-chlorophenoxy) pyridin-4-yl) methanamine **(Int-4)** (0.16 g, 0.68 mmol, 1.0 equiv) in dichloromethane (3.2 mL), triethylamine (0.2 g, 2.04 mmol, 3.0 equiv) and 1-acetylindoline-5-sulfonyl chloride **(Int-5)** (0.23 g, 0.88 mmol, 1.3 equiv) were added. The mixture was then stirred for 1 h at room temperature. The reaction mixture was poured into water, extracted with dichloromethane, dried over sodium sulfate, and concentrated under reduced pressure to give the crude compound which was purified by column chromatography using (SiO_2_, ethyl acetate:hexanes = 2:10 to 4:10) to give 1-acetyl-*N*-((2-(2-chlorophenoxy) pyridin-4-yl) methyl) indoline-5-sulfonamide **(MR44915)** (0.07 g, yield: 22.40%) as a white solid.

**LC-MS**: *m*/*z* = 458.0 [M + H]^+^.

^1^H NMR (400 MHz, DMSO-*d*_6_) δ 8.23 (s, 1H), 8.16 (d, J = 8.8 Hz, 1H), 7.99 (d, J = 5.2 Hz, 1H), 7.62 – 7.39 (m, 3H), 7.41 (t, J = 7.8 Hz, 1H), 7.31– 7.23 (m, 2H), 7.04 (d, J = 5.3 Hz, 1H), 6.97 (s, 1H), 4.17 (t, J = 8.7 Hz, 2H), 4.09 (d, J = 6.3 Hz, 2H), 3.19 (t, J = 6.5 Hz, 2H), 2.21 (s, 3H).

^13^C NMR (101 MHz, DMSO-*d*_6_) δ 170.22, 163.04, 152.23, 149.77, 146.71, 134.91, 133.58, 130.81, 128.97, 127.03, 124.74, 123.77, 118.49, 115.75, 109.41, 49.16, 45.25, 27.52, 24.60

## Data Availability

Experimental 3D protein structure coordinates are deposited in the Protein Data Bank under accession codes: 6N31, 6PBG, 6VYC, 7KLJ, 7KQQ, 7KYX, 7M3X, 7MT1, 7RUO, 7STY, 7SUL, 7UFV, 8D30, 8D41, 8DYS, 8F8E, 8SHJ, 8T5I, 8T55, 8UWP, 8W3V. Authors will release the atomic coordinates upon article publication. Protein expression clones have been deposited to Addgene (and are listed in Table S3 as reference). Purification protocols and associated data for all WDR proteins manufactured are deposited.^31^ Any additional information required to reanalyze the data reported in this paper is available from the lead author upon request.

## Supporting Information Available

**Figure S1** illustrates PubMed-extracted research activity on WDRs, and an illustration of the sequence identity of WDR containing proteins (the raw data for Figure S1 is in Tables S1 and S2); **Figure S2** illustrates the ligand-bound, 3D structures of four WDR proteins in the literature (from before we started the project); **Figure S3** gives some background on the DEL selections; It also provides some metrics on the binding affinity of the ligands and their similarity to the DEL selections; **Figure S4** shows an overlay of a new 3D structure for WDR91 and RAB7A with our structures with small molecule ligands; **Figure S5** has additional binding data for the WDR5 small molecule ligand; **Figure S6** shows more detailed data on HDX-MS of WDR12 in the presence of a small molecule ligand; **Figure S7** shows the full 1H-15N HSQC spectra that relates to Figure 5; **Figure S8** shows a dose–response SPR experiment of MT34329 binding to DCAF1-WDR; **Figure S9** shows examples of the quality control for capturing proteins on solid phase matrix before DEL selections; **Figure S10** shows the LC-UV/MS as a measure of quality control of small molecules; **Table S6** lists the apo and ligand-bound 3D structures generated during this project; **Table S7** relates to Table S6 and contains the related data collection and refinement statistics (PDF)
**Tables S1** and **S2** comprise the data used to generate the illustrations in Figure S1 (XLSX) (CSV)
**Table S3** lists all constructs deposited to Addgene (XLSX)
**Table S4** gives a brief overview of protein purification (with details in ref (31), DOI: 10.5281/zenodo.10655441) (XLSX)
**Table S5** lists buffer conditions for SPR and melting temperatures for 30 WDRs (XLSX)
**Table S8** lists the DLID scores and relates to Figure 1d (XLSX)
**Table S9** lists conditions for DEL selections (XLSX)
**Table S10** relates to the ML models used to generate the predictions (XLSX)
**Table S11** comprises the Molecular Formula Strings of each predicted compound, the associated binding and solubility data (CSV)
**Table S12** lists cellular melting temperatures and the nanoBiT reagents (XLSX)
**Table S13** lists the nanoBRET assay components (XLSX)
Full wwPDB X-ray Structure Validation Report (PDF)

## Abbreviations

BLI: Biolayer Interferometry
DEL: DNA Encoded Chemical Library
DEL-ML: DNA Encoded Chemical Library followed by Machine Learning
DLID: drug-like density
DSF: Differential Scanning Fluorimetry
FP: Fluorescence Polarization
GCNN: Graph Convolutional Neural Network
HDX: Hydrogen–Deuterium eXchange
HTS: High-Throughput Screening
ML: Machine Learning
NanoBRET: NanoLuciferase Bioluminescence Resonance Energy Transfer
NMR: Nuclear Magnetic Resonance
PPI: Protein–Protein Interaction
PROTAC: Proteolysis Targeting Chimera
SPR: Surface Plasmon Resonance
*T*_m_: melting temperature
WDR: Tryptophan-Aspartate Repeat.
