# Supplementary material for: A Target Class Ligandability Evaluation of WD40 Repeat-Containing Proteins
Source: J Med Chem. 2024 Nov 4;68(2):1092–112. doi: 10.1021/acs.jmedchem.4c02010 (PMC11770632; doi:10.1021/acs.jmedchem.4c02010)
Supplement: Supplementary file 1 — jm4c02010_si_001.pdf [file jm4c02010_si_001.pdf]

Supplementary Information

A target class ligandability evaluation of WD40 repeat-containing proteins

Suzanne Ackloo[1], Fengling Li[1], Magda Szewczyk[1], Almagul Seitova[1], Peter Loppnau[1], Hong Zeng[1], Jin Xu[2], Shabbir Ahmad[1], Yelena A Arnautova[3], AJ Baghaie[3], Serap Beldar[1], Albina Bolotokova[1], Paolo A Centrella[4], Irene Chau[1], Matthew A Clark[4], John W Cuozzo[3,5], Saba Dehghani-Tafti[1], Jeremy S Disch[3,5], Aiping Dong[1], Antoine Dumas[3], Jianwen A. Feng[2], Pegah Ghiabi[1], Elisa Gibson[1], Justin Gilmer[2], Brian Goldman[3,5], Stuart R Green[1], Marie-Aude Guié[4], John P Guilinger[4], Nathan Harms[3], Oleksandra Herasymenko[1], Scott Houliston[6], Ashley Hutchinson[1], Steven Kearnes[2,5], Anthony D Keefe[4], Serah W Kimani[1], Trevor Kramer[3,5], Maria Kutera[1], Haejin A Kwak[1], Cristina Lento[8], Yanjun Li[1], Jenny Liu[4], Joachim Loup[9], Raquel AC Machado[1], Christopher J Mulhern[3,4], Sumera Perveen[1], Germanna L Righetto[1], Patrick Riley[2,5], Suman Shrestha[1], Eric A Sigel[3], Madhushika Silva[1], Michael D. Sintchak[7], Belinda L Slakman[3,4,5], Rhys D Taylor[4], James Thompson[2], Wen Torng[2], Carl Underkoffler[3,5], Moritz von Rechenberg[3,5], Ryan T Walsh[4], Ian Watson[2], Derek J Wilson[8], Esther Wolf[8], Manisha Yadav[1], Aliakbar K Yazdi[1], Junyi Zhang[3,5], Ying Zhang[4], Vijayaratnam Santhakumar[1], Aled M Edwards[1], Dalia Barsyte-Lovejoy[1,10], Matthieu Schapira[1,10], Peter J Brown[1], Levon Halabelian*[1,10], Cheryl H Arrowsmith*[1,6]

[1]Structural Genomics Consortium, University of Toronto, 101 College St, Toronto, ON M5G 1L7, Canada
[2]Google, 1600 Amphitheatre Parkway, Mountain View, CA 94043, USA
[3]ZebiAI Inc., 100 Beaver St., Waltham MA 02435, USA
[4]X-Chem Inc., 100 Beaver St., Waltham MA 02435, USA
[5]Relay Therapeutics, 399 Binney St., Cambridge MA 02139, USA
[6]Princess Margaret Cancer Centre, University of Toronto, Toronto, ON M5G 2M9, Canada
[7]Civetta Therapeutics, 10 Wilson Rd, Cambridge, MA 02138, USA
[8]Department of Chemistry, York University, Toronto, ON M3J 1P3, Canada
[9]Department of Chemistry, University of Toronto, ON M5S 3H6, Canada
[10]Department of Pharmacology and Toxicology, University of Toronto, Toronto, ON M5S 1A8, Canada

*Corresponding authors

Contents

Figure S1. WDR research activity and a sequence identity matrix	S3
Figure S2. Ligand-bound structures of WDRs	S4
Figure S3. Positive training examples from DEL selections, and plots of PTEs relative to binding affinity	S5
Figure S4. WDR91 and Rab7 interact in a unique pocket relative to MR45279 and MR46654	S6
Figure S5. DSF and BLI data for WDR5 DEL-ML hit	S7
Figure S6. Hydrogen-deuterium exchange mass spectrometry to characterize binding of MR44915 to WDR12	S8
Figure S7. The full 1H-15N HSQC spectra of 100 μM 15N-lablelled DNMT3A-PWWP	S9
Figure S8. SPR data of DNMT3A hit MT34329 binds to DCAF1-WDR	S10
Figure S9: Examples of protein capture quality control used for DEL selections	S11
Figure S10: LC-UV/MS of compounds	S12
Table S6. List of high-resolution 3D structures of WDR domain proteins	S21
Table S7a. Data collection and refinement statistics for CORO6A, WDR55, WDR41	S22
Table S7b. Data collection and refinement statistics for COPB2, KIF21A, WDR12	S23
Table S7c. Data collection and refinement statistics for SEC31A, COPA, UTP15	S24
Table S7d. Data collection and refinement statistics for CORO1C, RBBP7, EIF2A	S25
Table S7e. Data collection and refinement statistics for PAFAH1B1, COPB2 with ligand, WDR5 with ligand	S26
Table S7f. Data collection and refinement statistics for SETDB1 with ligand	S27

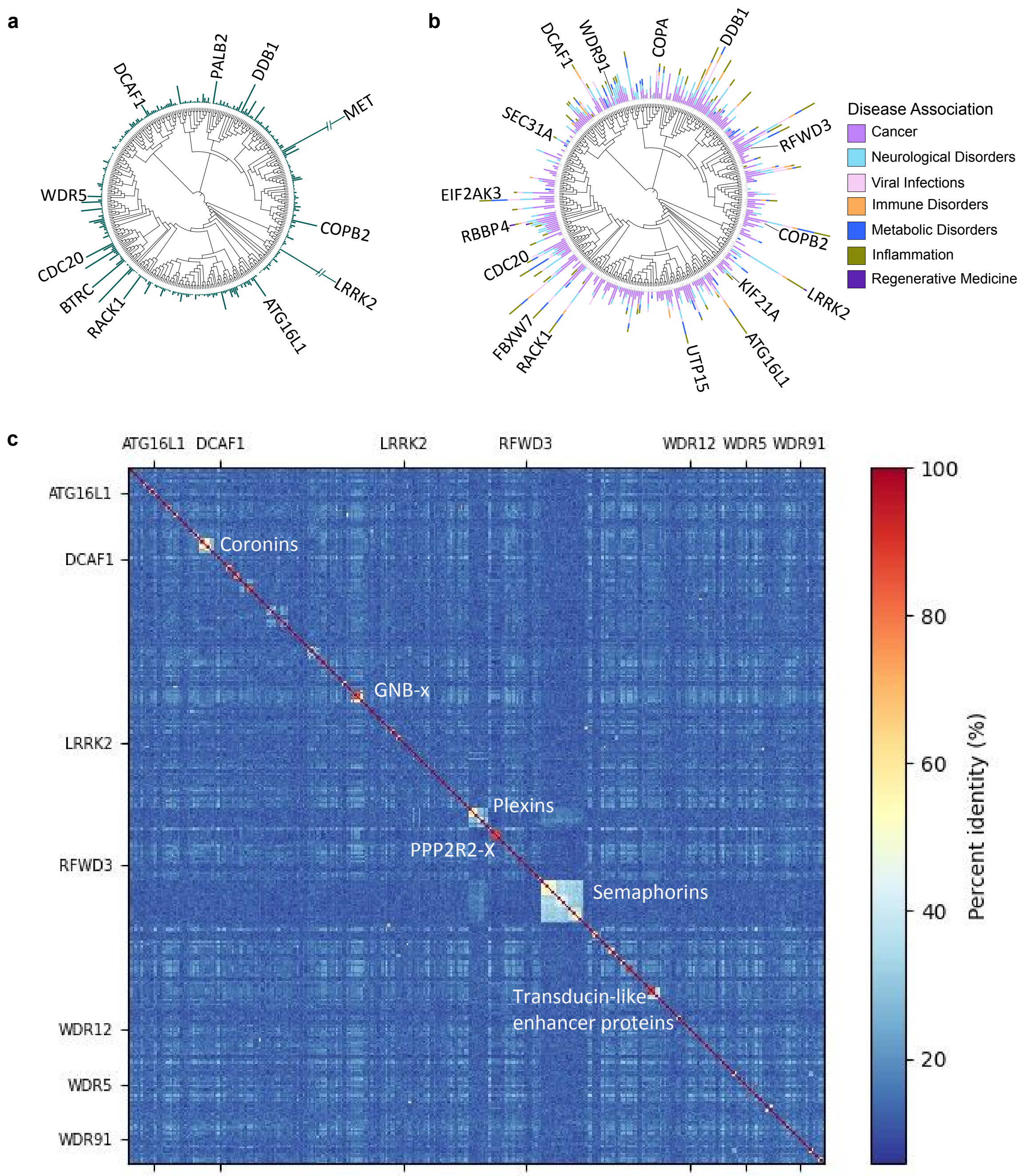

Fig S1 | WDR research activity and a sequence identity matrix. A phylogenetic tree of the WDR containing protein family where each line represents a different protein. **(a)** The length of the line represents the number of publications that report on WDR containing proteins indexed in PubMed (but excludes publications that list >30 genes), (**b**) The length represents $\log_2$ of the number of publications indexed in PubMed associating the protein with a specific disease category (color-coded), (**c**) A pairwise sequence alignment of the human WDR domains listed on https://doi.org/10.5281/zenodo.10655441 was conducted using ICM-Pro 3.9-3a (Molsoft, San Diego). The resulting distance matrix is provided, with color-coding reflecting sequence identity as shown on the right. Proteins are ordered alphabetically as in **Table S8**. Some protein subfamilies have high sequence identity and are highlighted as clusters along the diagonal.

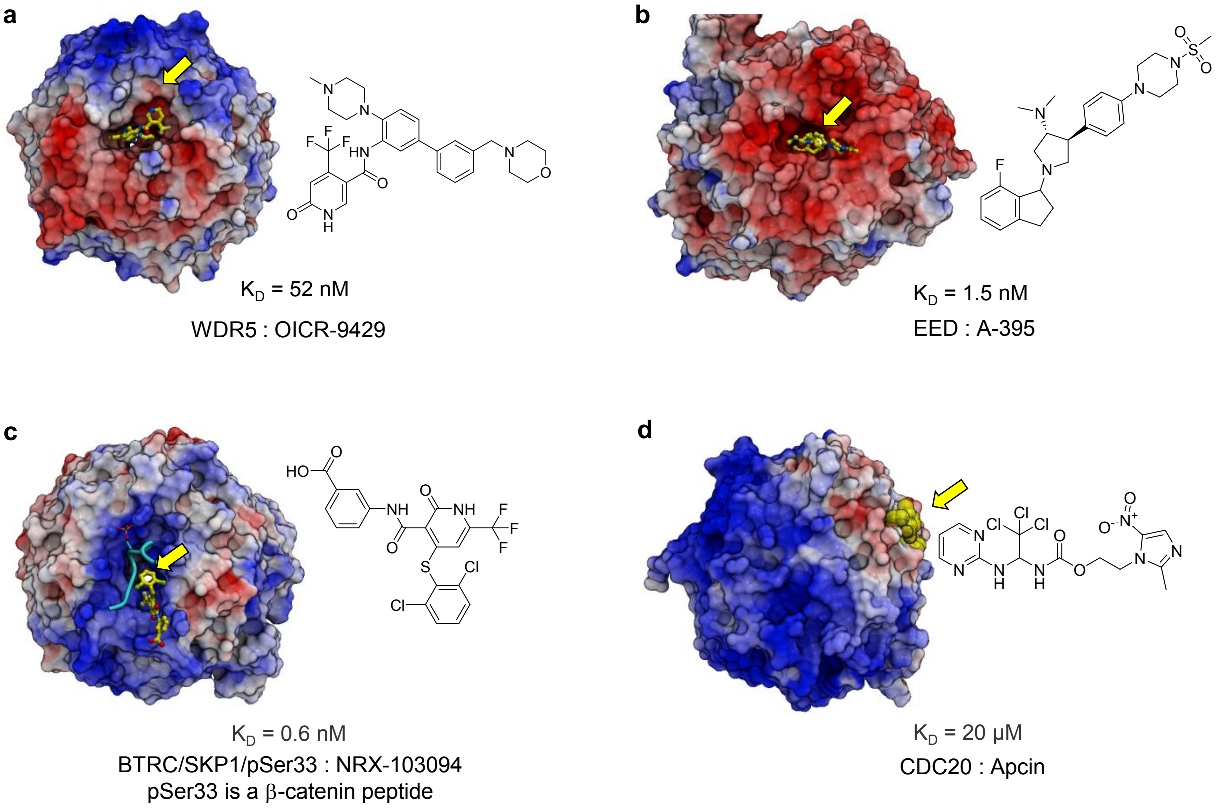

Fig S2 | Ligand-bound structures of WDRs. (**a**) WDR5 in complex with OICR-9429 (PDB: 4QL1)[1], (**b**) EED in complex with A-395 (PDB: 5K0M)[2], (**c**) BTRC/SKP1/*pSer33 in complex with NRX-103094 (PDB: 6M91)[3], (**d**) CDC20 in complex with apcin (PDB: 4N14)[4]. The 3D structures illustrate the diversity of electrostatic potential distribution around the protein surface and in the central pocket. Small molecule ligands are displayed in yellow in the 3D structure, and to the right are the chemical structures with the associated binding (K_D) constants. Surface electrostatic potential with colors saturating below −5 kcal/e.u. charge units (red) and above +5 kcal/e.u. charge units (blue). *pSer33 is a β-catenin peptide.

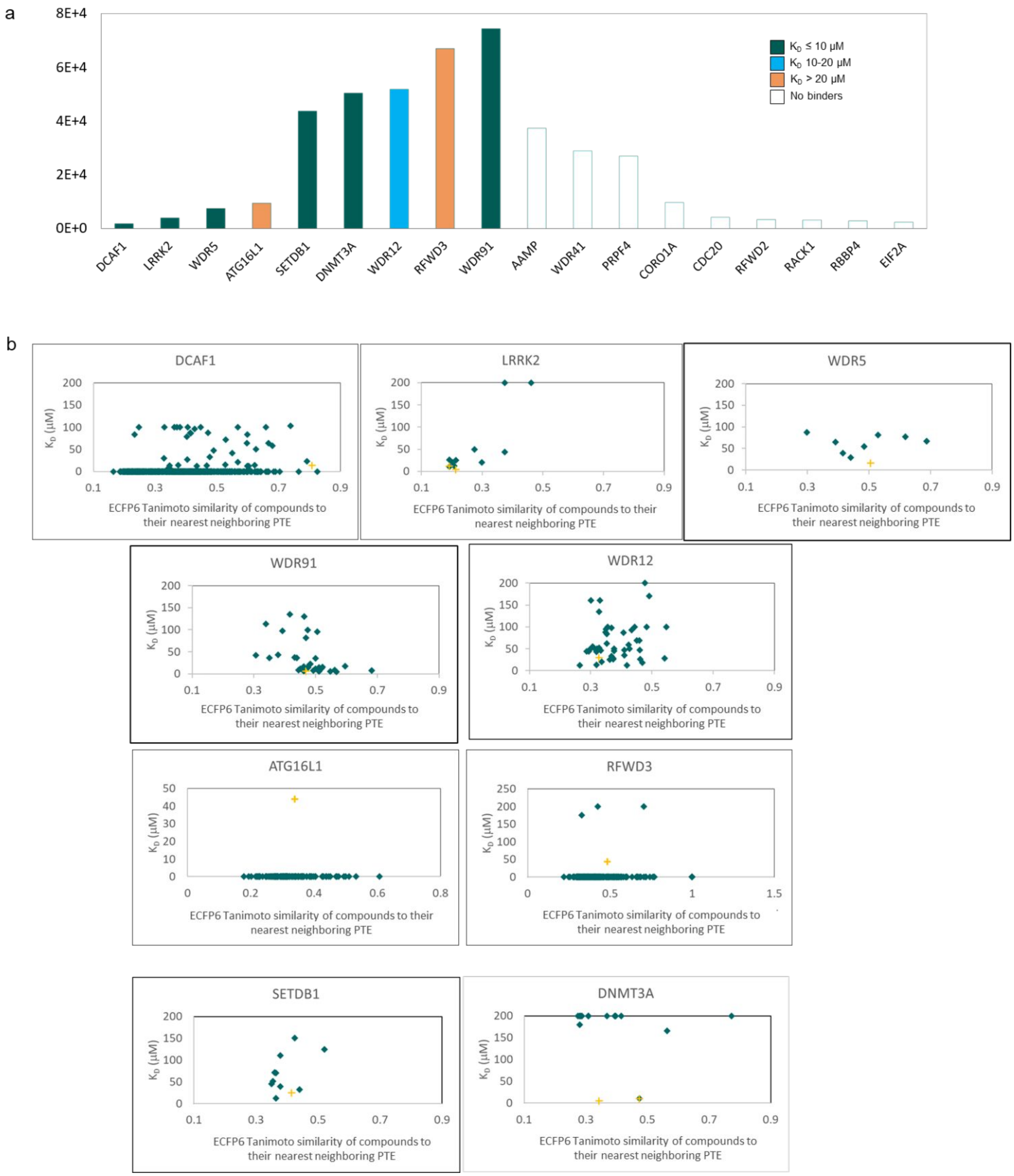

Figure S3: Positive training examples from DEL selections, and plots of PTEs relative to binding affinity. (a) The number of positive training examples for each target; see also Tables S8 and S9, and (b) plots of K_D (determined by SPR) against the ECFP6 Tanimoto similarity of predictions to their nearest neighboring PTE. The (yellow) plus sign on the plots represent the primary hit for each target.

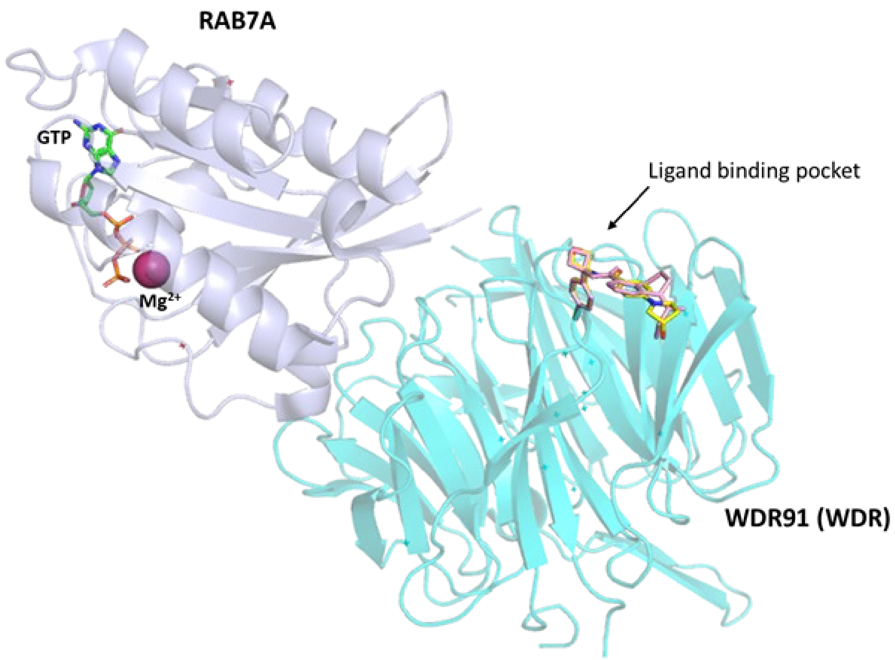

Fig. S4| WDR91 and Rab7 interact in a unique pocket relative to MR45279 and MR46654. Superposition of WDR91-Rab7 complex (PBD ID 8kb8, Rab7- purple, WDR91- cyan) with the WDR91-compound complexes (PDB ID 8SHJ, 8T55) bound to a non-covalent compound MR45279 (yellow sticks) and its covalent analogue MR46654 (pink sticks) in the side pocket of the WDR domain, showing that the ligand binding pocket is not overlapped with the Rab7 binding interface.

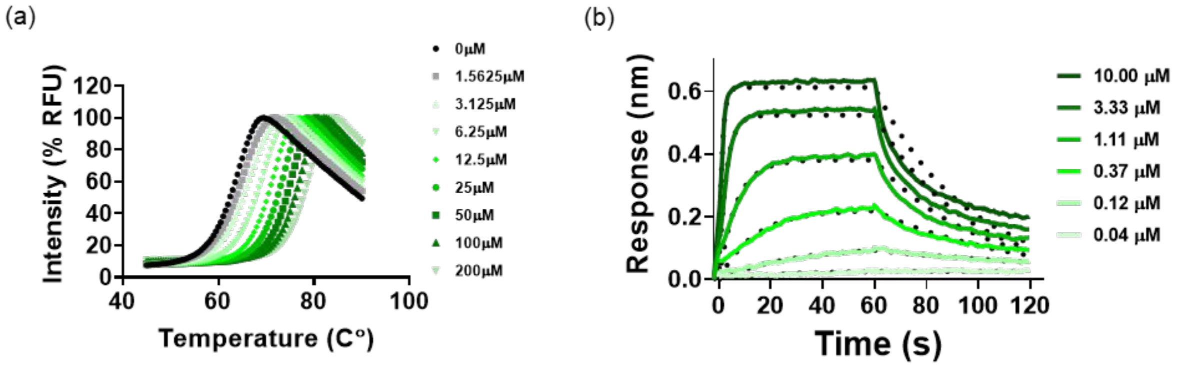

Fig. S5 | DSF and BLI data for WDR5. (a) Thermal stability of WDR5 was assessed in the presence and absence of MR44397 using differential scanning fluorimetry (DSF). Effect of MR44397 binding on the stability of WDR5 was measured by DSF at 0.1 mg/mL WDR5 in the presence of 0-200 μM of MR44397. **(b)** BLI analysis of the binding of MR44397 to WDR5. A representative sensorgram (solid green) is shown with the globally fitted (black dots) with a K_D value of 497 nM, k_{on} = 0.6 × 10^{+5} $M^{-1}s^{-1}$; k_{off} = 2.8 × 10^{-2} s^{-1}. All experiments were performed in triplicate.

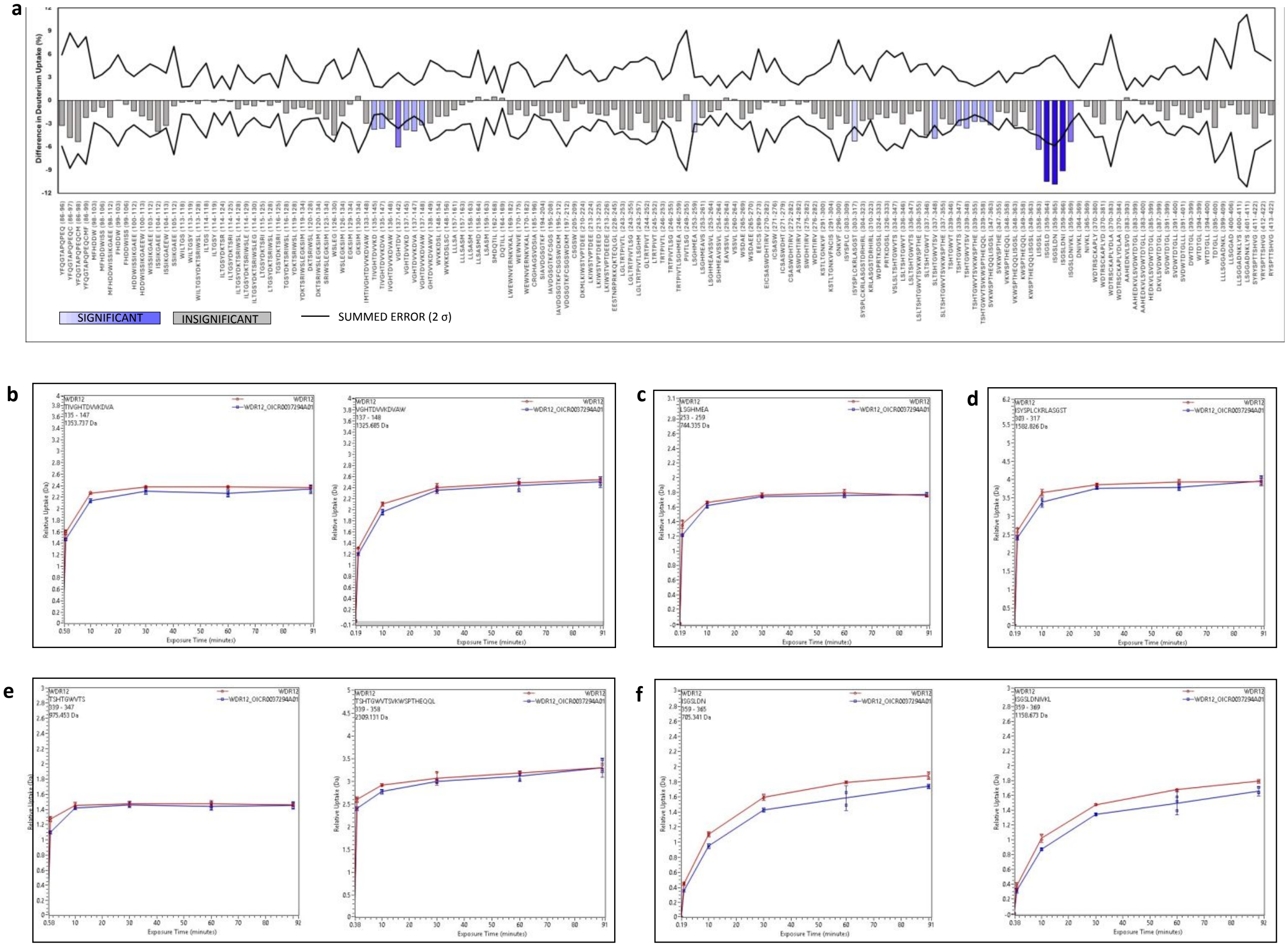

Fig. S6 | Hydrogen-deuterium exchange mass spectrometry to characterize binding of MR44915 to WDR12.
(**a**) Summed differences in the deuterium uptake of WDR12 in the presence of MR44915. WDR12 peptide sequences are plotted against the extent of *D* uptake perturbation (%). To be statistically significant, the cumulative D uptake perturbation (%) needed to exceed the cumulative propagated error (2σ).
HDX-MS kinetic plots of WDR12 peptides spanning residues (**b**) 135-148, (**c**) 253-259, (**d**) 303-317, (**e**) 339-358, and (**f**) 359-369. The deuterium uptake of WDR12 (red) and WDR12+MR44915 1:20 (blue) was tracked across 1, 10, 30, 60, and 90 mins. Divergence in the red and blue lines indicate perturbations in conformational dynamics due to binding MR44915. Complexation caused (**b**)-(**e**) to experience decreased deuterium uptake early in the time course whereas residues 359-369 (in (**f**)) had persistently reduced uptake over the time course.

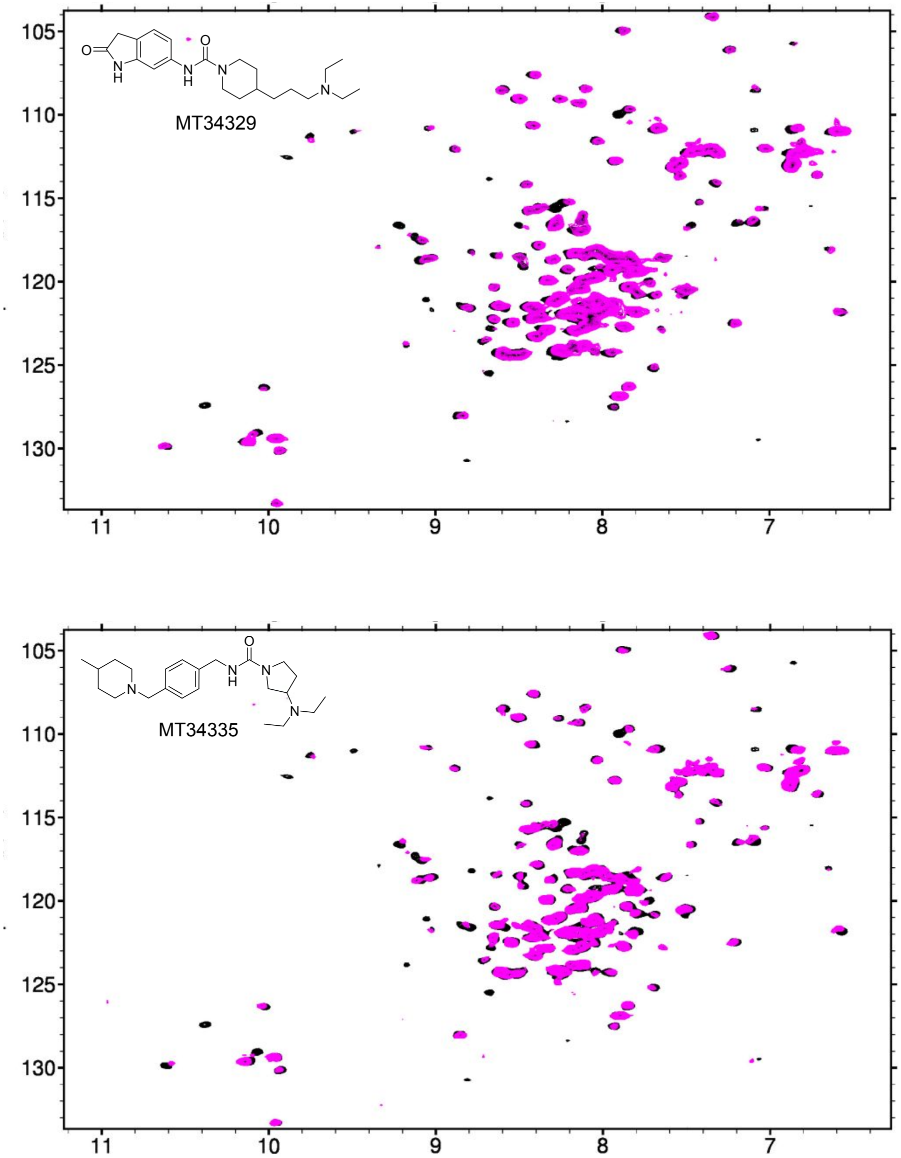

Figure S7. The full ^{1}H-^{15}N HSQC spectra of 100 μM ^{15}N-lablelled DNMT3A-PWWP (●) and ^{15}N-lablelled DNMT3A-PWWP with 500 μM of (a) MT34329 (●) and (b) MT34335. In the presence of MT34329 and MT34335 we observe general broadening and/or chemical shift perturbations.

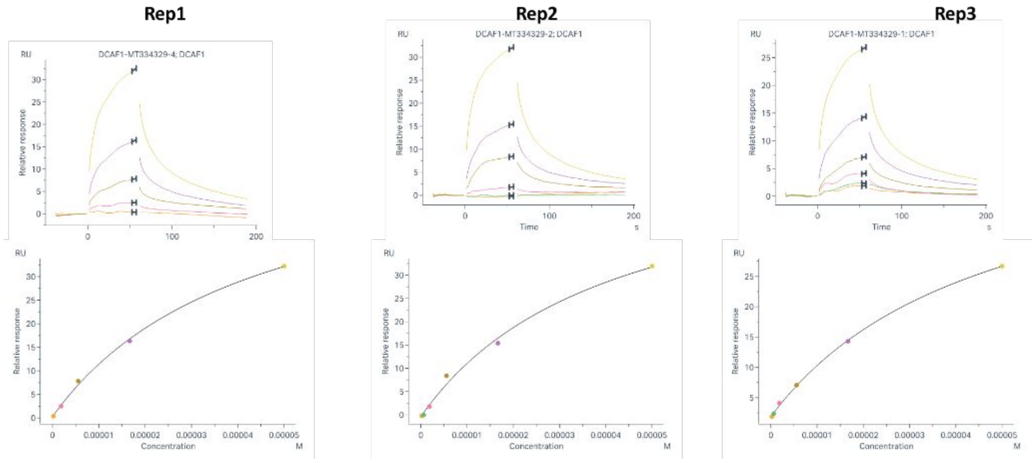

Fig. S8| The DNMT3A hit MT34329 binds to DCAF1-WDR with a K_D value of 44 μM.

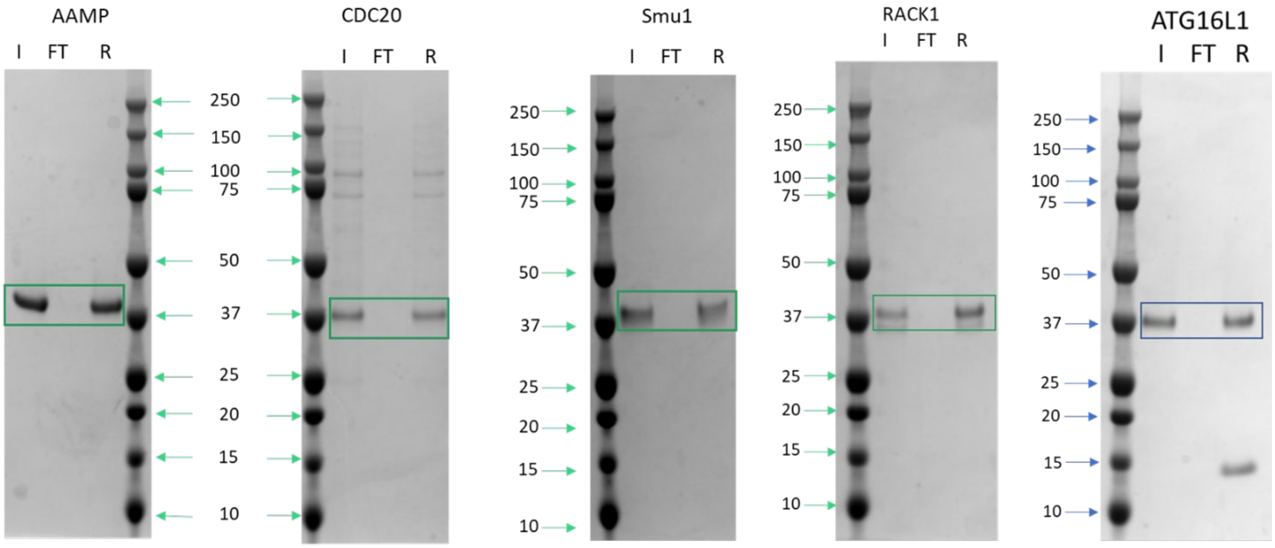

Fig S9. Examples of protein capture QC used for DEL selections. I = input; FT = flow-through; R = recovery

Compound	SMILES	LC-UV/MS	Supporting Information
MR43378	CCCC(C(N1CCc2c(C1)ccs2)=O)NC(c1cccc(Cn2ccnc2)c1)=O	x	x
MR44397	CCC[C@H](NC(C1=CC=CC(CN2C=CN=C2)=C1)=O)C(N3CCC4=C(C=CS4)C3)=O	x	x
DR02380	c1ccc(cc1)c1cc(C(Nc2nnc(C(F)(F)F)s2)=O)n(c2ccccc2)n1	x	x
DR02034	CC(C(Nc1nc(cs1)c1ccccc1)=O)OC(C1C(C=C(C)N(c2ccccc2)N=1)=O)=O	x	x
MR40903	CC(N1CCc2cc(ccc12)S(NCc1ccnc(c1)Oc1ccccc1F)(=O)=O)=O	x	x
MT34329	CCN(CC)CCCC1CCN(CC1)C(Nc1ccc2CC(Nc2c1)=O)=O	x	x
MR45279	C1CN(CC1O)c1ccc(cc1)C(NC1(COC1)c1ccc(cc1)[Cl])=O	x	x
MR43625	CCN(CC)CCNC(c1cc(ccc1[Cl])NC(N1CCC(C1)N(CC)CC)=O)=O	x	x

SETDB1: MR43625

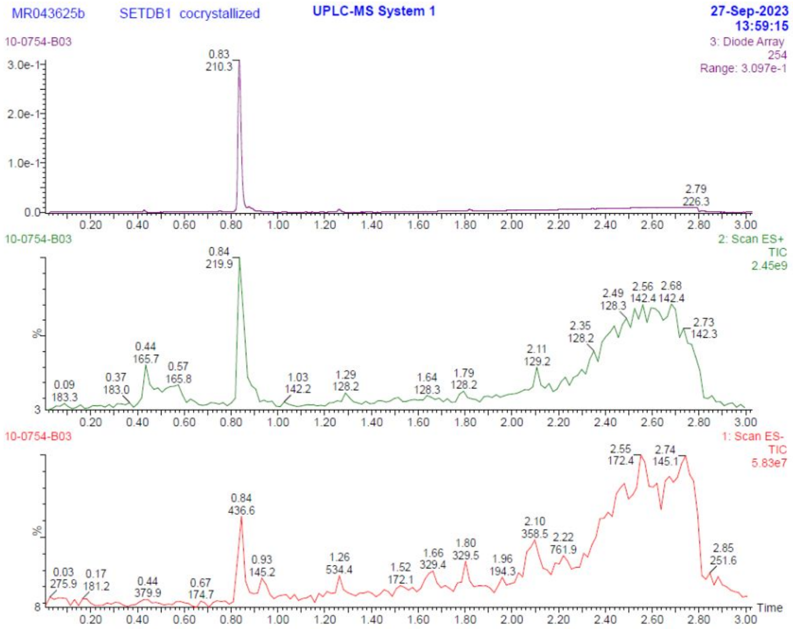

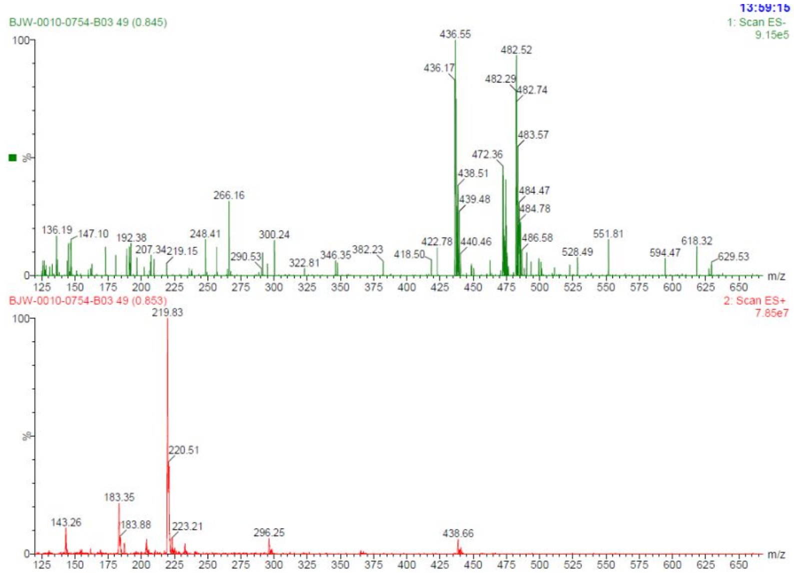

DNMT3A: MR34329

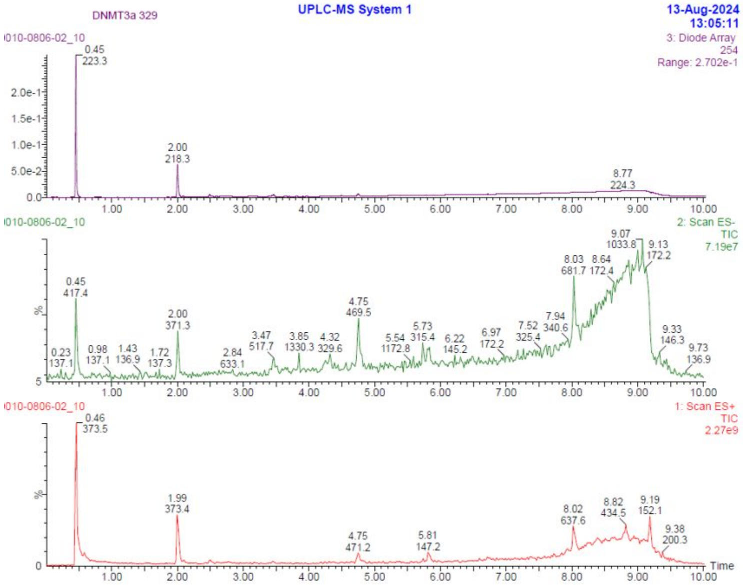

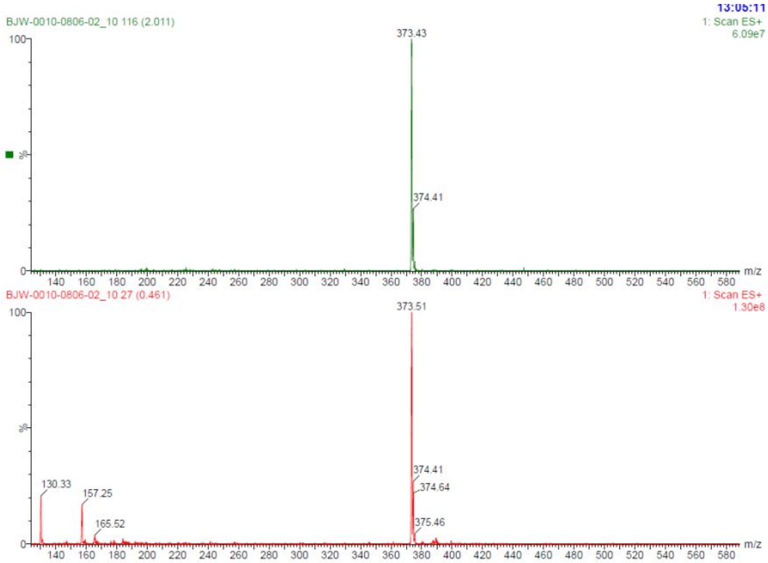
13:05:11
BJW-0010-0806-02_10 116 (2.011)
1: Scan ES+
6.09e7
373.43
374.41
m/z
BJW-0010-0806-02_10 27 (0.461)
1: Scan ES+
1.30e8
373.51
374.41
374.64
375.46
130.33
157.25
165.52
m/z

WDR12: MR40903

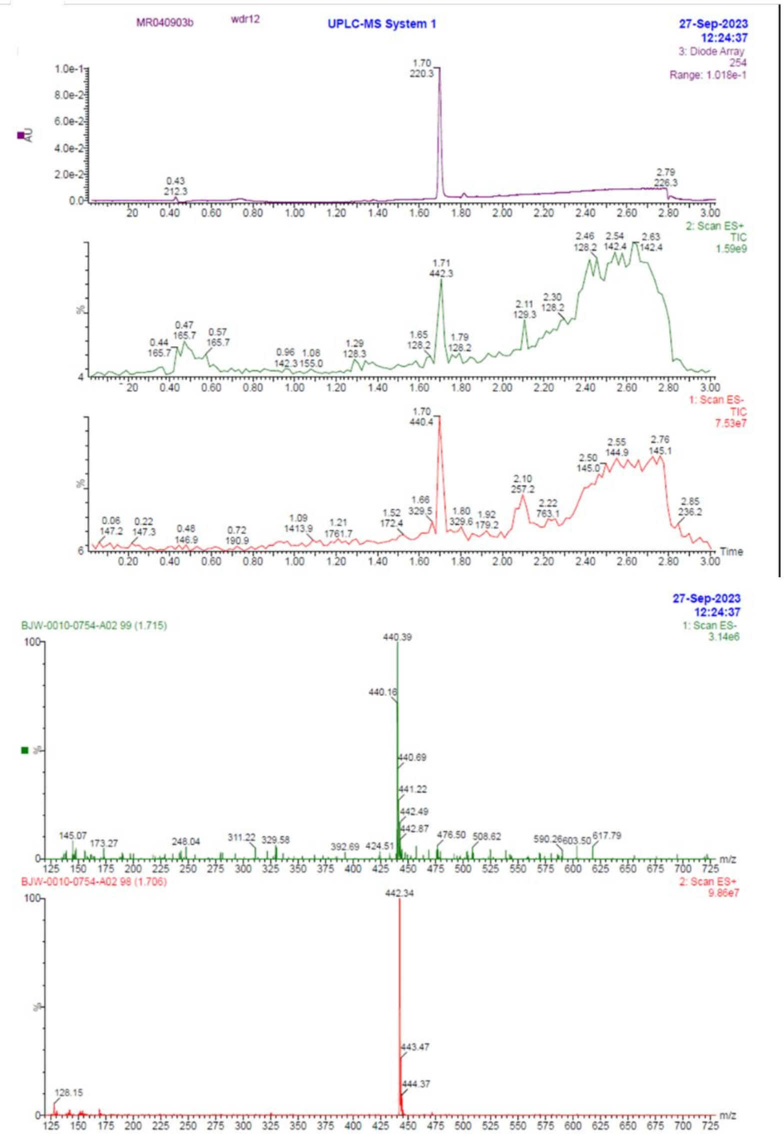

WDR5: MR43378

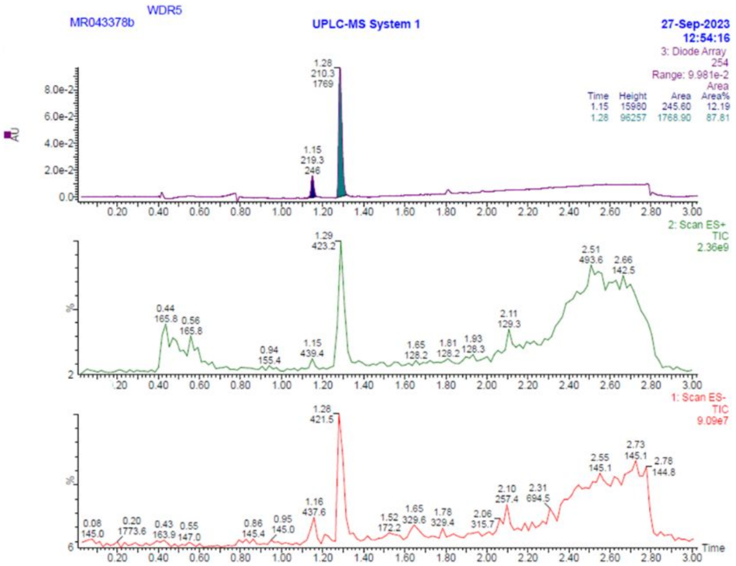

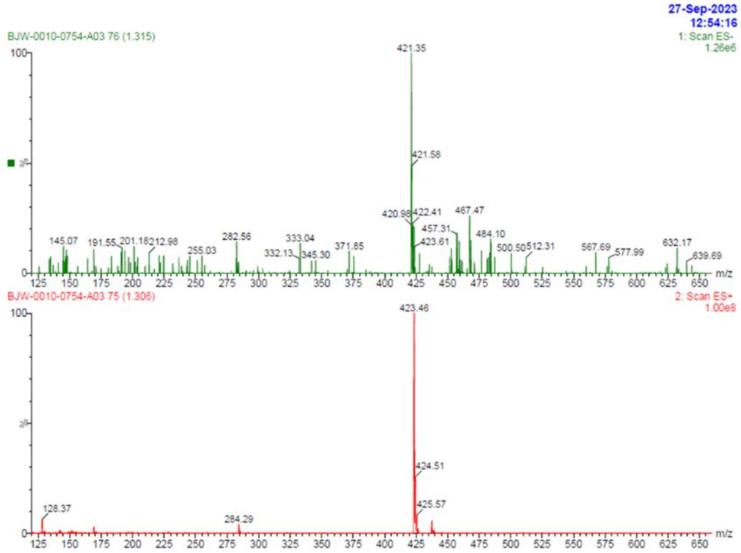

WDR5: MR44397

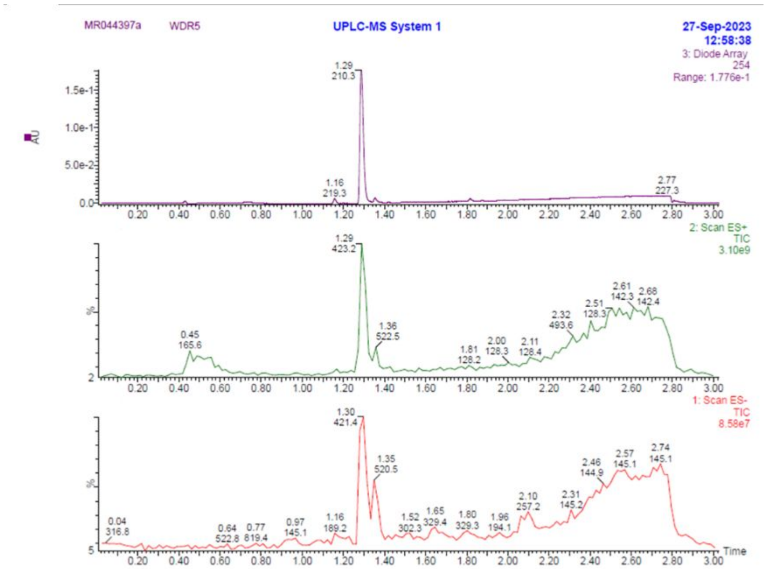

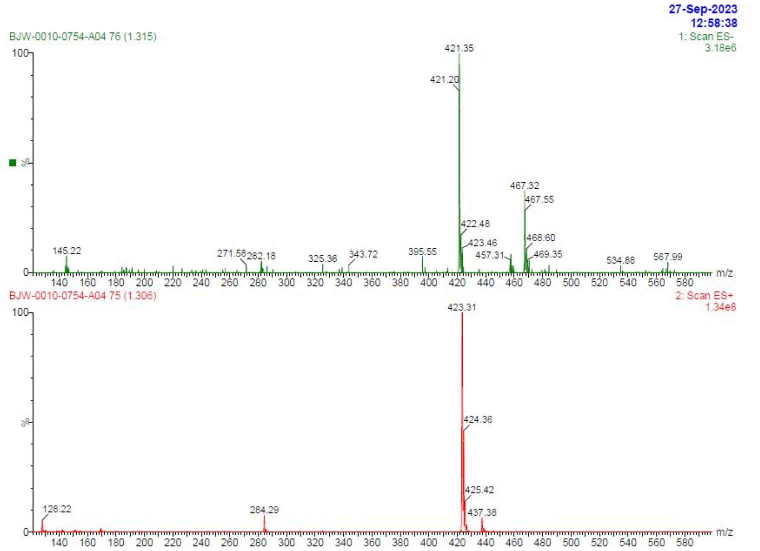

LRRK2: DR02034

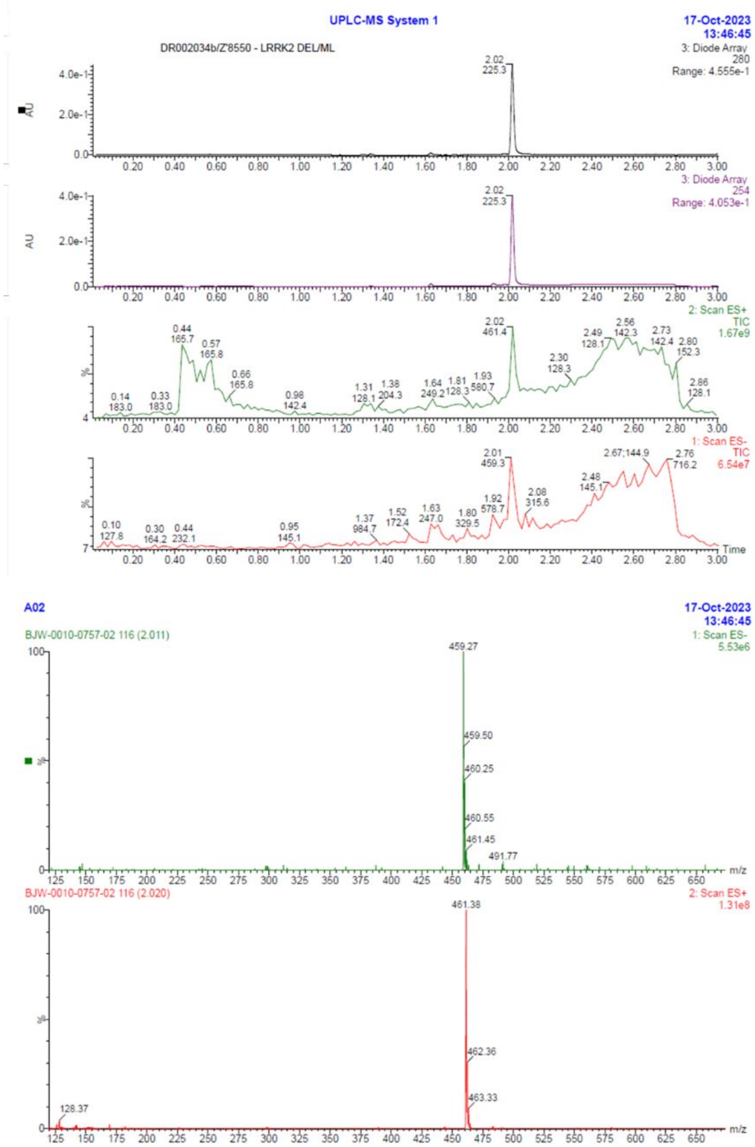

LRRK2: DR02380

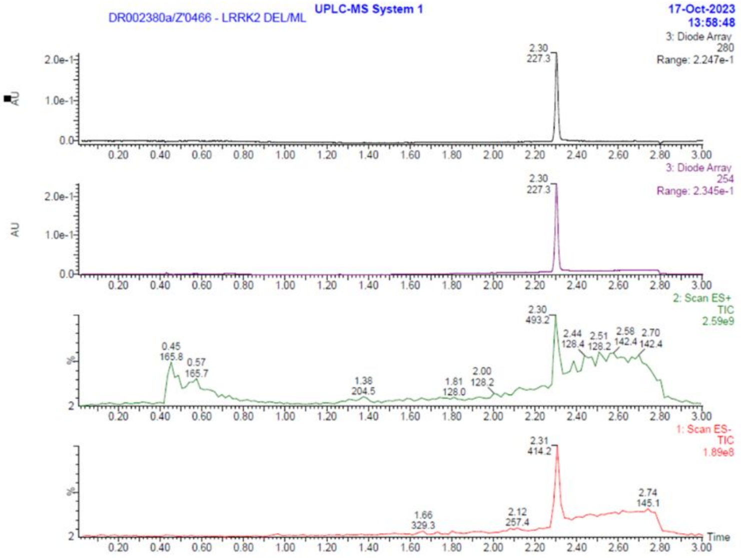

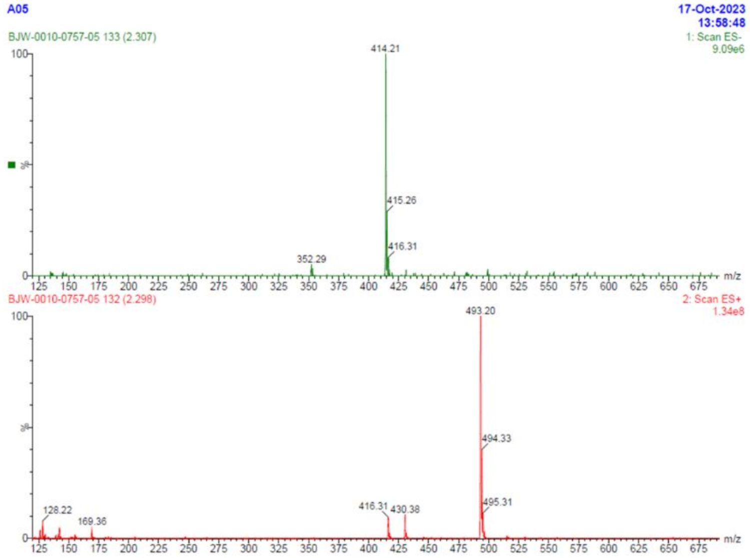

WDR91: MR45279

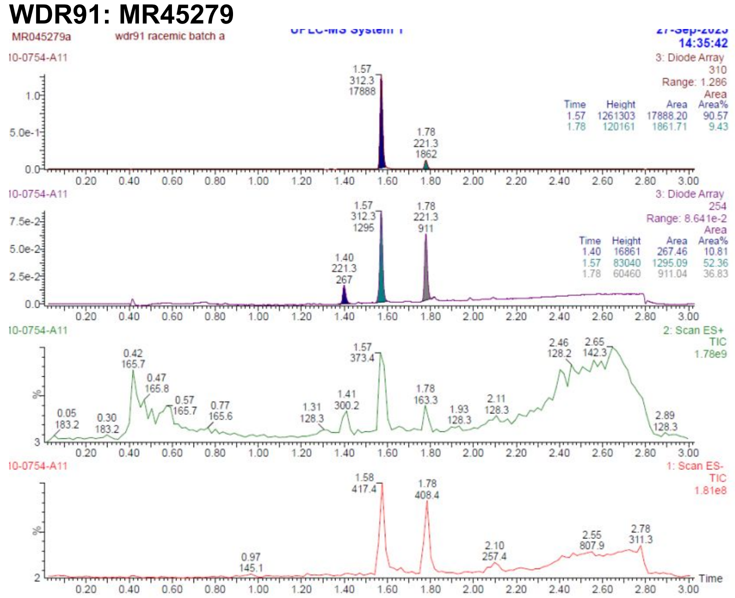

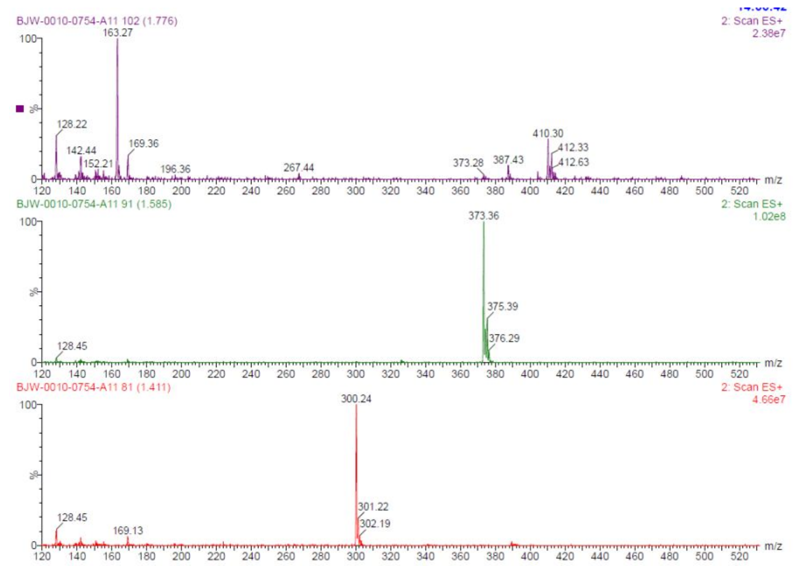

Fig. S10 | HPLC-UV/MS for hits and Figs. 2-6.

Table S6. List of high-resolution 3D structures of WDR domain proteins

Description	PDB ID	Comment
COPA	6PBG	Apo
COPB2 WD-domain in complex with OICR-6254	8D41	with ligand
COPB2 WD-domains	8D30	Apo
CORO1C	7STY	Apo
CORO6	7KYX	Apo
DCAF1 in complex with OICR-6766	7UFV	with ligand
DCAF1 in complex with OICR-8268	8F8E	with ligand
eIF2A	8DYS	Apo
KIF21A	7KLJ	Apo
PAFAH1B1	7MT1	Apo
RBBP7	7M3X	Apo
SEC31A	7SUL	Apo
SETDB1 Tandem Tudor domain in complex with MR46747	8UWP	with ligand
UTP15	7RUO	Apo
WDR12	6N31	Apo
WDR41	8W3V	Apo
WDR5 in complex with MR44397	8T51	with ligand
WDR55	7KQQ	Apo
WDR91	6VYC	Apo
WDR91 in complex with MR45279	8SHJ	with ligand
WDR91 in complex with MR46654	8T55	with ligand

Table S7a. Data collection and refinement statistics for CORO6A, WDR55, WDR41

	**CORO6**	**WDR55**	**WDR41**
PDB ID	7KYX	7KQQ	8W3V
Wavelength (Å)	0.97918	0.97911	0.97911
Resolution range (Å)	50.00-1.63 (1.66-1.63) *	48.75-1.80 (1.84-1.80) *	35.0-2.20(2.24-2.20) *
Space group	$P2_12_12_1$	$P2_1$	C2
Unit cell (Å)	50.5, 80.8, 96.4	79.0, 58.6, 88.2	229.1, 86.0,52.6
Total reflections	454557	234127	219586
Unique reflections	50011(2387)	72091(4411)	49100(1891)
Multiplicity	9.1(7.4)	3.2(3.4)	4.5(3.7)
Completeness (%)	99.5(97.4)	96.9 (99.5)	95.3(74.6)
Mean I/sigma(I)	24.8(1.73)	8.2(1.4)	22.1(1.4)
Wilson B-factor	15.1	29.9	49.2
R-merge	0.092(0.942)	0.071(0.567)	0.066(0.953)
R-meas	0.098(1.012)	0.085(0.673)	0.075(1.094)
R-pim	0.032(0.360)	0.045(0.365)	0.034(0.532)
CC1/2	0.997(0.790)	0.995(0.705)	0.994(0.692)
Reflections used in refinement	48957	68592	46626
Reflections used for R-free	989	3484	2466
R-work	0.174	0.188	0.216
R-free	0.210	0.233	0.253
Number of non-hydrogen atoms			
Macromolecules	3140	4704	4921
Ligands	n/a	n/a	n/a
Solvent	250	294	39
Protein residues			
RMS (bonds)	0.007	0.011	0.006
RMS (angles)	1.449	1.644	0.862
Ramachandran favoured (%)	97.2	97.0	93.8
Ramachandran allowed (%)	100.0	100.0	98.8
Ramachandran outliers (%)	0.0	0.0	1.2
Rotamer outliers (%)	0.60	0.40	0.82
Clash score	2.58	1.71	4.93
Average B-factor			
Macromolecules	22.2	35.7	69.4
Ligands	n/a	n/a	n/a
Solvent	31.3	40.8	60.8

*Statistics for the highest-resolution shell are shown in parentheses.

Table S7b. Data collection and refinement statistics for COPB2, KIF21A, WDR12

	COPB2 WD-domain	KIF21A	WDR12
PDB ID	8D30	7KLJ	6N31
Wavelength (Å)	0.97918	1.54178	0.97918
Resolution range (Å)	50.00-2.40 (2.44-2.40) *	50.00-1.50 (1.55-1.52) *	76.00-2.60 (2.71-2.60) *
Space group	$P2_12_12_1$	$P2_1$	$P2_12_12_1$
Unit cell (Å)	67.1, 134.1, 144.3	55.3, 67.1, 79.9	52.7, 87.1, 155.9
Total reflections	288205	600649	65932
Unique reflections	51291 (2439)	89050(3875)	21949(8091)
Multiplicity	5.6(4.8)	6.7(3.9)	3.0(3.0)
Completeness (%)	99.0(97.1)	99.3(87.1)	96.7(95.7)
Mean I/sigma(I)	21.0(1.45)	35.8(2.98)	7.4(1.1)
Wilson B-factor	48.8	15.2	64.8
R-merge	0.080 (1.005)	0.073(0.424)	0.109(1.028)
R-meas	0.089 (1.123)	0.078(0.487)	0.133(1.252)
R-pim	0.037(0.486)	0.029(0.233)	0.046 (0.523)
CC1/2	0.998(0.496)	0.997(0.821)	0.993(0.476)
Reflections used in refinement	51241	87717	21880
Reflections used for R-free	2516	1307	1046
R-work	0.219	0.170	0.203
R-free	0.247	0.185	0.233
Number of non-hydrogen atoms			
Macromolecules	8483	5004	4707
Ligands	n/a	n/a	n/a
Solvent	80	556	n/a
Protein residues			
RMS (bonds)	0.010	0.007	0.010
RMS (angles)	1.19	1.439	1.30
Ramachandran favoured (%)	94.6	95.7	96.1
Ramachandran allowed (%)	99.6	100.0	100.0
Ramachandran outliers (%)	0.4	0.0	0.0
Rotamer outliers (%)	1.50	0.92	3.23
Clash score	3.29	1.62	4.78
Average B-factor			
Macromolecules	61.4	20.1	49.7
Ligands	n/a	n/a	n/a
Solvent	50.4	29.8	n/a

*Statistics for the highest-resolution shell are shown in parentheses.

Table S7c. Data collection and refinement statistics for SEC31A, COPA, UTP15

	SEC31A	COPA	UTP15
PDB ID	7SUL	6PBG	7RUO
Wavelength (Å)	0.97918	1.5418	1.54178
Resolution range (Å)	50.00-2.40(2.44-2.40) *	50.00-1.72(1.75-1.72) *	42.66-1.80 (1.84-1.80) *
Space group	$P2_1$	P2	P1
Unit cell (Å)	51.2, 156.7, 85.2	34.6, 85.5, 47.9	58.3, 59.1, 61.9
Total reflections	167966	170533	125959
Unique reflections	49159(1994)	26521(977)	57233(3260)
Multiplicity	3.4(3.0)	6.4(4.1)	2.2(2.2)
Completeness (%)	94.2(75.0)	93.6(69.3)	93.7(90.0)
Mean I/sigma(I)	13.1(1.46)	21.3(2.08)	4.7(2.0)
Wilson B-factor	38.6	14.6	12.2
R-merge	0.116(0.781)	0.088(0.472)	0.108(0.464)
R-meas	0.136(0.917)	0.096(0.532)	0.145(0.625)
R-pim	0.069(0.473)	0.037(0.236)	0.096(0.415)
CC1/2	0.985(0.700)	0.962(0.833)	0.982(0.672)
Reflections used in refinement	49130	25722	54327
Reflections used for R-free	986	740	2896
R-work	0.189	0.171	0.231
R-free	0.237	0.218	0.263
Number of non-hydrogen atoms			
Macromolecules	9956	2491	4723
Ligands	n/a	n/a	n/a
Solvent	69	220	292
Protein residues			
RMS (bonds)	0.010	0.008	0.007
RMS (angles)	1.20	1.352	1.490
Ramachandran favoured (%)	95.8	95.1	96.0
Ramachandran allowed (%)	100.0	100.0	100.0
Ramachandran outliers (%)	0.0	0.0	0.0
Rotamer outliers (%)	3.94	0.75	2.14
Clash score	3.56	2.04	4.7
Average B-factor			
Macromolecules	53.3	18.9	16.2
Ligands	n/a	n/a	n/a
Solvent	44.2	27.1	20.4

*Statistics for the highest-resolution shell are shown in parentheses.

Table S7d. Data collection and refinement statistics for CORO1C, RBBP7, EIF2A

	**CORO1C**	**RBBP7**	**eIF2A**
PDB ID	7STY	7M3X	8DYS
Wavelength (Å)	0.97918	0.97918	0.97918
Resolution range (Å)	50.00-2.00(2.03-2.00) *	50.00-1.46(1.49-1.46) *	47.67-1.80(1.84-1.80) *
Space group	$P3_12_1$	$P2_12_12_1$	$P2_12_12_1$
Unit cell (Å)	127.2, 127.2, 59.2	44.7, 88.8, 97.2	54.3, 84.8, 99.3
Total reflections	145933	727132	326278
Unique reflections	35890(1520)	68121(3354)	42642(2481)
Multiplicity	4.1(3.4)	10.7(8.1)	7.7(7.9)
Completeness (%)	95.1(81.8)	99.9(99.8)	98.8(98.1)
Mean I/sigma(I)	18.9(1.3)	31.0(1.71)	13.5(3.6)
Wilson B-factor	40.0	10.4	23.0
R-merge	0.096(0.877)	0.074(0.777)	0.108(0.808)
R-meas	0.110(1.021)	0.078(0.829)	0.117(0.864)
R-pim	0.053(0.513)	0.023(0.286)	0.043(0.304)
CC1/2	0.990(0.507)	0.997(0.805)	0.995(0.832)
Reflections used in refinement	34775	64741	40514
Reflections used for R-free	1097	3307	2087
R-work	0.179	0.170	0.200
R-free	0.221	0.193	0.228
Number of non-hydrogen atoms			
Macromolecules	3028	3085	3178
Ligands	n/a	n/a	n/a
Solvent	167	423	199
Protein residues			
RMS (bonds)	0.010	0.007	0.008
RMS (angles)	1.397	1.446	1.436
Ramachandran favoured (%)	97.4	98.0	97.1
Ramachandran allowed (%)	100.0	99.5	100.0
Ramachandran outliers (%)	0.0	0.5	0.0
Rotamer outliers (%)	0.94	0.88	0.60
Clash score	3.87	2.65	2.56
Average B-factor			
Macromolecules	43.8	16.2	28.3
Ligands	n/a	n/a	n/a
Solvent	44.5	25.9	33.0

*Statistics for the highest-resolution shell are shown in parentheses.

Table S7e. Data collection and refinement statistics for PAFAH1B1, COPB2 with ligand, WDR5 with ligand

	**PAFAH1B1**	**COPB2+OICR-6254**	**WDR5+MR44397**
PDB ID	7MT1	8D41	8T5I
Wavelength (Å)	0.97918	1.54178	0.97918
Resolution range (Å)	36.02-1.30 (1.32-1.30) *	48.87-2.00 (2.05-2.00) *	50.00-1.70(1.73-1.70) *
Space group	$P2_1$	$P2_1$	C2
Unit cell (Å)	36.7, 68.6, 69.5	57.0, 91.1, 58.7	101.4, 86.4, 80.8
Total reflections	464783	160563	416078
Unique reflections	82396(3866)	38534(2656)	72369(3548)
Multiplicity	5.6(5.3)	4.2(3.9)	5.7(5.4)
Completeness (%)	99.2(94.4)	96.1(90.1)	95.9(95.1)
Mean I/sigma(I)	22.5(3.1)	11.3(3.0)	15.8(1.85)
Wilson B-factor	12.6	10.1	15.9
R-merge	0.034(0.477)	0.112(0.534)	0.135(0.982)
R-meas	0.037(0.531)	0.128(0.617)	0.149(1.087)
R-pim	0.015(0.228)	0.062(0.305)	0.062(0.456)
CC1/2	1.000(0.862)	0.993(0.779)	0.983(0.658)
Reflections used in refinement	78418	36560	70899
Reflections used for R-free	3953	1919	1467
R-work	0.177	0.171	0.197
R-free	0.197	0.220	0.232
Number of non-hydrogen atoms			
Macromolecules	2554	4871	4731
Ligands	n/a	24	60
Solvent	258	351	440
Protein residues			
RMS (bonds)	0.007	0.010	0.007
RMS (angles)	1.431	1.276	1.398
Ramachandran favoured (%)	95.3	95.6	95.4
Ramachandran allowed (%)	99.7	99.8	100.0
Ramachandran outliers (%)	0.3	0.2	0.0
Rotamer outliers (%)	0.0	0.93	0.95
Clash score	1.19	4.28	3.46
Average B-factor			
Macromolecules	16.5	20.3	18.7
Ligands	n/a	30.8	18.2
Solvent	28.7	25.0	27.5

*Statistics for the highest-resolution shell are shown in parentheses.

Table S7f. Data collection and refinement statistics for SETDB1 with ligand

	**SETDB1+MR46747**
PDB ID	8UWP
Wavelength (Å)	0.97918
Resolution range (Å)	50.00-1.76(1.79-1.76) *
Space group	$P2_12_12$
Unit cell (Å)	63.3, 141.7, 55.5
Total reflections	403142
Unique reflections	49143(2422)
Multiplicity	8.2(7.7)
Completeness (%)	99.0(98.8)
Mean I/sigma(I)	27.6(1.83)
Wilson B-factor	25.3
R-merge	0.077(0.940)
R-meas	0.082(1.002)
R-pim	0.028(0.341)
CC1/2	0.996(0.881)
Reflections used in refinement	47896
Reflections used for R-free	1202
R-work	0.199
R-free	0.242
Number of non-hydrogen atoms	
Macromolecules	3529
Ligands	60
Solvent	201
Protein residues	
RMS (bonds)	0.009
RMS (angles)	1.363
Ramachandran favoured (%)	97.4
Ramachandran allowed (%)	100.0
Ramachandran outliers (%)	0.0
Rotamer outliers (%)	1.11
Clash score	5.21
Average B-factor	
Macromolecules	49.7
Ligands	34.7
Solvent	37.5

*Statistics for the highest-resolution shell are shown in parentheses.

References

1. Grebien, F.; Vedadi, M.; Getlik, M.; Giambruno, R.; Grover, A.; Avellino, R.; Skucha, A.; Vittori, S.; Kuznetsova, E.; Smil, D.; Barsyte-Lovejoy, D.; Li, F.; Poda, G.; Schapira, M.; Wu, H.; Dong, A.; Senisterra, G.; Stukalov, A.; Huber, K. V. M.; Schonegger, A.; Marcellus, R.; Bilban, M.; Bock, C.; Brown, P. J.; Zuber, J.; Bennett, K. L.; Al-Awar, R.; Delwel, R.; Nerlov, C.; Arrowsmith, C. H.; Superti-Furga, G., Pharmacological targeting of the Wdr5-MLL interaction in C/EBPalpha N-terminal leukemia. *Nature chemical biology* **2015,** *11* (8), 571-578.
2. He, Y.; Selvaraju, S.; Curtin, M. L.; Jakob, C. G.; Zhu, H.; Comess, K. M.; Shaw, B.; The, J.; Lima-Fernandes, E.; Szewczyk, M. M.; Cheng, D.; Klinge, K. L.; Li, H. Q.; Pliushchev, M.; Algire, M. A.; Maag, D.; Guo, J.; Dietrich, J.; Panchal, S. C.; Petros, A. M.; Sweis, R. F.; Torrent, M.; Bigelow, L. J.; Senisterra, G.; Li, F.; Kennedy, S.; Wu, Q.; Osterling, D. J.; Lindley, D. J.; Gao, W.; Galasinski, S.; Barsyte-Lovejoy, D.; Vedadi, M.; Buchanan, F. G.; Arrowsmith, C. H.; Chiang, G. G.; Sun, C.; Pappano, W. N., The EED protein-protein interaction inhibitor A-395 inactivates the PRC2 complex. *Nat Chem Biol* **2017,** *13* (4), 389-395.
3. Simonetta, K. R.; Taygerly, J.; Boyle, K.; Basham, S. E.; Padovani, C.; Lou, Y.; Cummins, T. J.; Yung, S. L.; von Soly, S. K.; Kayser, F.; Kuriyan, J.; Rape, M.; Cardozo, M.; Gallop, M. A.; Bence, N. F.; Barsanti, P. A.; Saha, A., Prospective discovery of small molecule enhancers of an E3 ligase-substrate interaction. *Nat Commun* **2019,** *10* (1), 1402.
4. Sackton, K. L.; Dimova, N.; Zeng, X.; Tian, W.; Zhang, M.; Sackton, T. B.; Meaders, J.; Pfaff, K. L.; Sigoillot, F.; Yu, H.; Luo, X.; King, R. W., Synergistic blockade of mitotic exit by two chemical inhibitors of the APC/C. *Nature* **2014,** *514* (7524), 646-9.
