# Supplementary material for: A Target Class Ligandability Evaluation of WD40 Repeat-Containing Proteins
Source: J Med Chem. 2024 Nov 4;68(2):1092–112. doi: 10.1021/acs.jmedchem.4c02010 (PMC11770632; doi:10.1021/acs.jmedchem.4c02010)
Supplement: Supplementary file 13 — jm4c02010_si_013.pdf [file jm4c02010_si_013.pdf]

Full wwPDB X-ray Structure Validation Report (i)

Nov 4, 2021 – 04:03 PM EDT

PDB ID	:	7RUO
Title	:	Crystal structure of human UTP15
Authors	:	Dehghani-Tafti, S.; Dong, A.; Zeng, H.; Hutchinson, A.; Seitova, A.; Loppnau, P.; Arrowsmith, C.H.; Edwards, A.M.; Halabelian, L.; Structural Genomics Consortium (SGC)
Deposited on	:	2021-08-17
Resolution	:	1.80 Å(reported)

This is a Full wwPDB X-ray Structure Validation Report for a publicly released PDB entry.

We welcome your comments at *validation@mail.wwpdb.org*
A user guide is available at
https://www.wwpdb.org/validation/2017/XrayValidationReportHelp
with specific help available everywhere you see the (i) symbol.

The following versions of software and data (see references (i)) were used in the production of this report:

MolProbity	:	4.02b-467
Xtriage (Phenix)	:	1.13
EDS	:	2.23.2
Percentile statistics	:	20191225.v01 (using entries in the PDB archive December 25th 2019)
Refmac	:	5.8.0158
CCP4	:	7.0.044 (Gargrove)
Ideal geometry (proteins)	:	Engh & Huber (2001)
Ideal geometry (DNA, RNA)	:	Parkinson et al. (1996)
Validation Pipeline (wwPDB-VP)	:	2.23.2

1 Overall quality at a glance (i)

The following experimental techniques were used to determine the structure:
X-RAY DIFFRACTION

The reported resolution of this entry is 1.80 Å.

Percentile scores (ranging between 0-100) for global validation metrics of the entry are shown in the following graphic. The table shows the number of entries on which the scores are based.

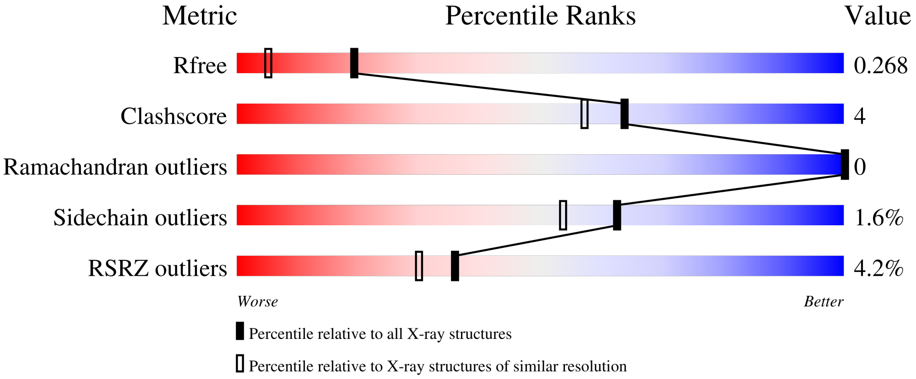

Metric	Whole archive (#Entries)	Similar resolution (#Entries, resolution range(Å))
R_{free}	130704	5950 (1.80-1.80)
Clashscore	141614	6793 (1.80-1.80)
Ramachandran outliers	138981	6697 (1.80-1.80)
Sidechain outliers	138945	6696 (1.80-1.80)
RSRZ outliers	127900	5850 (1.80-1.80)

The table below summarises the geometric issues observed across the polymeric chains and their fit to the electron density. The red, orange, yellow and green segments of the lower bar indicate the fraction of residues that contain outliers for >=3, 2, 1 and 0 types of geometric quality criteria respectively. A grey segment represents the fraction of residues that are not modelled. The numeric value for each fraction is indicated below the corresponding segment, with a dot representing fractions <=5% The upper red bar (where present) indicates the fraction of residues that have poor fit to the electron density. The numeric value is given above the bar.

Mol	Chain	Length	Quality of chain
1	A	388	4% / 72% 5% • 22%
1	B	388	3% / 70% 7% 23%

The following table lists non-polymeric compounds, carbohydrate monomers and non-standard residues in protein, DNA, RNA chains that are outliers for geometric or electron-density-fit criteria:

Mol	Type	Chain	Res	Chirality	Geometry	Clashes	Electron density
2	UNX	A	1001	-	-	-	X
2	UNX	B	401	-	-	-	X
2	UNX	B	402	-	-	-	X

2 Entry composition (i)

There are 3 unique types of molecules in this entry. The entry contains 5020 atoms, of which 0 are hydrogens and 0 are deuteriums.

In the tables below, the ZeroOcc column contains the number of atoms modelled with zero occupancy, the AltConf column contains the number of residues with at least one atom in alternate conformation and the Trace column contains the number of residues modelled with at most 2 atoms.

- Molecule 1 is a protein called U3 small nucleolar RNA-associated protein 15 homolog.

Mol	Chain	Residues	Atoms					ZeroOcc	AltConf	Trace
1	A	301	Total<br>2364	C<br>1504	N<br>407	O<br>445	S<br>8	0	4	0
1	B	300	Total<br>2359	C<br>1503	N<br>401	O<br>447	S<br>8	0	5	0

There are 36 discrepancies between the modelled and reference sequences:

Chain	Residue	Modelled	Actual	Comment	Reference
A	-17	MET	-	initiating methionine	UNP Q8TED0
A	-16	HIS	-	expression tag	UNP Q8TED0
A	-15	HIS	-	expression tag	UNP Q8TED0
A	-14	HIS	-	expression tag	UNP Q8TED0
A	-13	HIS	-	expression tag	UNP Q8TED0
A	-12	HIS	-	expression tag	UNP Q8TED0
A	-11	HIS	-	expression tag	UNP Q8TED0
A	-10	SER	-	expression tag	UNP Q8TED0
A	-9	SER	-	expression tag	UNP Q8TED0
A	-8	GLY	-	expression tag	UNP Q8TED0
A	-7	ARG	-	expression tag	UNP Q8TED0
A	-6	GLU	-	expression tag	UNP Q8TED0
A	-5	ASN	-	expression tag	UNP Q8TED0
A	-4	LEU	-	expression tag	UNP Q8TED0
A	-3	TYR	-	expression tag	UNP Q8TED0
A	-2	PHE	-	expression tag	UNP Q8TED0
A	-1	GLN	-	expression tag	UNP Q8TED0
A	0	GLY	-	expression tag	UNP Q8TED0
B	-17	MET	-	initiating methionine	UNP Q8TED0
B	-16	HIS	-	expression tag	UNP Q8TED0
B	-15	HIS	-	expression tag	UNP Q8TED0
B	-14	HIS	-	expression tag	UNP Q8TED0
B	-13	HIS	-	expression tag	UNP Q8TED0
B	-12	HIS	-	expression tag	UNP Q8TED0
B	-11	HIS	-	expression tag	UNP Q8TED0

Continued on next page...

Continued from previous page...

Chain	Residue	Modelled	Actual	Comment	Reference
B	-10	SER	-	expression tag	UNP Q8TED0
B	-9	SER	-	expression tag	UNP Q8TED0
B	-8	GLY	-	expression tag	UNP Q8TED0
B	-7	ARG	-	expression tag	UNP Q8TED0
B	-6	GLU	-	expression tag	UNP Q8TED0
B	-5	ASN	-	expression tag	UNP Q8TED0
B	-4	LEU	-	expression tag	UNP Q8TED0
B	-3	TYR	-	expression tag	UNP Q8TED0
B	-2	PHE	-	expression tag	UNP Q8TED0
B	-1	GLN	-	expression tag	UNP Q8TED0
B	0	GLY	-	expression tag	UNP Q8TED0

- Molecule 2 is UNKNOWN ATOM OR ION (three-letter code: UNX) (formula: X).

Mol	Chain	Residues	Atoms	ZeroOcc	AltConf
2	A	3	Total X<br>3 3	0	0
2	B	2	Total X<br>2 2	0	0

- Molecule 3 is water.

Mol	Chain	Residues	Atoms	ZeroOcc	AltConf
3	A	142	Total O<br>143 143	0	1
3	B	148	Total O<br>149 149	0	1

3 Residue-property plots ⓘ

These plots are drawn for all protein, RNA, DNA and oligosaccharide chains in the entry. The first graphic for a chain summarises the proportions of the various outlier classes displayed in the second graphic. The second graphic shows the sequence view annotated by issues in geometry and electron density. Residues are color-coded according to the number of geometric quality criteria for which they contain at least one outlier: green = 0, yellow = 1, orange = 2 and red = 3 or more. A red dot above a residue indicates a poor fit to the electron density (RSRZ > 2). Stretches of 2 or more consecutive residues without any outlier are shown as a green connector. Residues present in the sample, but not in the model, are shown in grey.

- Molecule 1: U3 small nucleolar RNA-associated protein 15 homolog

Chain A:

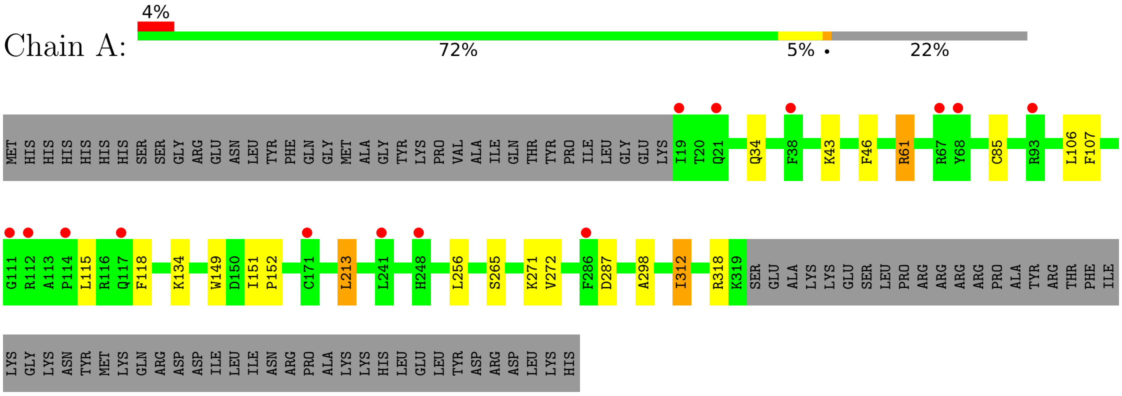

- Molecule 1: U3 small nucleolar RNA-associated protein 15 homolog

Chain B:

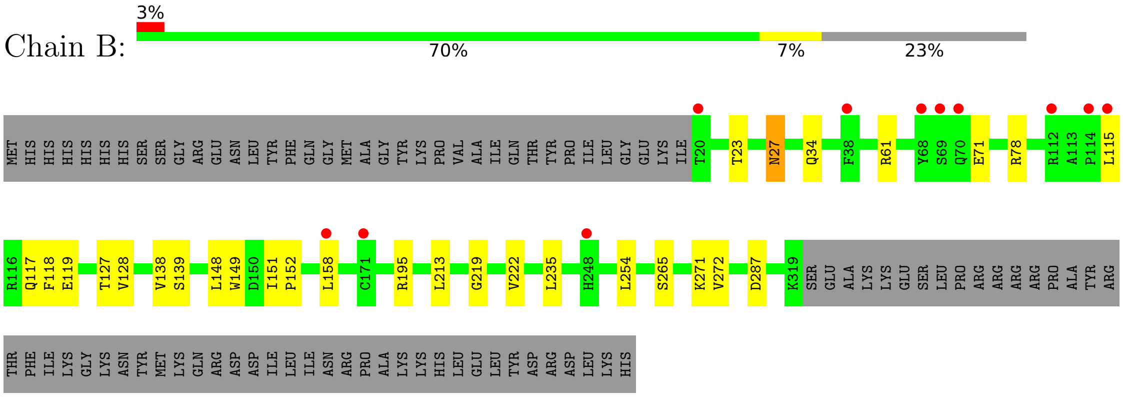

4 Data and refinement statistics (i)

Property	Value	Source
Space group	P 1	Depositor
Cell constants<br>a, b, c, α, β, γ	58.33Å 59.09Å 61.87Å<br>90.11° 66.82° 62.87°	Depositor
Resolution (Å)	42.69 – 1.80<br>42.66 – 1.80	Depositor<br>EDS
% Data completeness<br>(in resolution range)	93.6 (42.69-1.80)<br>93.6 (42.66-1.80)	Depositor<br>EDS
R_{merge}	0.11	Depositor
R_{sym}	(Not available)	Depositor
$< I/\sigma(I) >$ [1]	2.16 (at 1.79Å)	Xtriage
Refinement program	REFMAC 5.8.0258	Depositor
R, R_{free}	0.231 , 0.263<br>0.239 , 0.268	Depositor<br>DCC
R_{free} test set	2896 reflections (5.06%)	wwPDB-VP
Wilson B-factor (Å^2)	13.6	Xtriage
Anisotropy	0.323	Xtriage
Bulk solvent k_{sol}(e/Å^3), B_{sol}(Å^2)	0.34 , 37.8	EDS
L-test for twinning[2]	$< \|L\| > = 0.50$, $< L^2 > = 0.33$	Xtriage
Estimated twinning fraction	No twinning to report.	Xtriage
F_o,F_c correlation	0.92	EDS
Total number of atoms	5020	wwPDB-VP
Average B, all atoms (Å^2)	16.0	wwPDB-VP

Xtriage's analysis on translational NCS is as follows: *The analyses of the Patterson function reveals a significant off-origin peak that is 22.17 % of the origin peak, indicating pseudo-translational symmetry. The chance of finding a peak of this or larger height randomly in a structure without pseudo-translational symmetry is equal to 6.0223e-03. The detected translational NCS is most likely also responsible for the elevated intensity ratio.*

[1] Intensities estimated from amplitudes.

[2] Theoretical values of $< |L| >$, $< L^2 >$ for acentric reflections are 0.5, 0.333 respectively for untwinned datasets, and 0.375, 0.2 for perfectly twinned datasets.

5 Model quality (i)

5.1 Standard geometry (i)

Bond lengths and bond angles in the following residue types are not validated in this section: UNX

The Z score for a bond length (or angle) is the number of standard deviations the observed value is removed from the expected value. A bond length (or angle) with $|Z| > 5$ is considered an outlier worth inspection. RMSZ is the root-mean-square of all Z scores of the bond lengths (or angles).

Mol	Chain	Bond lengths		Bond angles	
		RMSZ	#|Z| >5	RMSZ	#|Z| >5
1	A	0.71	0/2419	0.82	0/3283
1	B	0.68	0/2414	0.84	0/3277
All	All	0.69	0/4833	0.83	0/6560

There are no bond length outliers.

There are no bond angle outliers.

There are no chirality outliers.

There are no planarity outliers.

5.2 Too-close contacts (i)

In the following table, the Non-H and H(model) columns list the number of non-hydrogen atoms and hydrogen atoms in the chain respectively. The H(added) column lists the number of hydrogen atoms added and optimized by MolProbity. The Clashes column lists the number of clashes within the asymmetric unit, whereas Symm-Clashes lists symmetry-related clashes.

Mol	Chain	Non-H	H(model)	H(added)	Clashes	Symm-Clashes
1	A	2364	0	2312	19	0
1	B	2359	0	2298	20	0
2	A	3	0	0	0	0
2	B	2	0	0	0	0
3	A	143	0	0	2	0
3	B	149	0	0	5	0
All	All	5020	0	4610	39	0

The all-atom clashscore is defined as the number of clashes found per 1000 atoms (including hydrogen atoms). The all-atom clashscore for this structure is 4.

All (39) close contacts within the same asymmetric unit are listed below, sorted by their clash

magnitude.

Atom-1	Atom-2	Interatomic distance (Å)	Clash overlap (Å)
1:A:61[B]:ARG:NH1	3:A:1101:HOH:O	2.12	0.81
1:B:195:ARG:NH1	3:B:501:HOH:O	2.18	0.76
1:A:151[A]:ILE:HB	1:A:152:PRO:HD3	1.69	0.74
1:B:78:ARG:HG2	1:B:115:LEU:HD11	1.70	0.73
1:B:271:LYS:HE3	1:B:287:ASP:OD2	1.93	0.69
1:A:271:LYS:HE2	3:B:638:HOH:O	1.98	0.62
1:B:117:GLN:CD	3:B:607[B]:HOH:O	2.41	0.60
1:B:23:THR:O	1:B:27:ASN:HB2	2.02	0.59
1:A:61[B]:ARG:HG2	1:A:61[B]:ARG:HH11	1.69	0.57
1:B:222[A]:VAL:HG13	1:B:254:LEU:HD13	1.88	0.56
1:B:148:LEU:HD23	1:B:158:LEU:HD13	1.88	0.55
1:B:78:ARG:NH1	1:B:117:GLN:OE1	2.40	0.55
1:A:106:LEU:HD11	1:A:151[A]:ILE:HD12	1.90	0.52
1:B:271:LYS:HE2	3:B:518:HOH:O	2.10	0.51
1:B:271:LYS:CE	3:B:518:HOH:O	2.58	0.51
1:B:151:ILE:HB	1:B:152:PRO:HD3	1.92	0.51
1:A:34:GLN:HG3	1:A:312:ILE:CD1	2.41	0.50
1:A:213[B]:LEU:HD11	1:A:256:LEU:HG	1.93	0.49
1:A:271:LYS:HE3	1:A:287:ASP:OD2	2.13	0.48
1:B:219:GLY:HA2	1:B:235:LEU:HG	1.97	0.47
1:B:118:PHE:HB3	1:B:149:TRP:CZ3	2.51	0.46
1:B:213:LEU:HB2	1:B:222[A]:VAL:CG1	2.46	0.45
1:A:34:GLN:HG3	1:A:312:ILE:HD13	1.98	0.45
1:A:34:GLN:OE1	1:A:34:GLN:N	2.49	0.45
1:B:213:LEU:CB	1:B:222[A]:VAL:HG12	2.46	0.45
1:A:151[B]:ILE:HB	1:A:152:PRO:HD3	2.00	0.43
1:B:128:VAL:HA	1:B:138:VAL:O	2.18	0.43
1:B:265:SER:O	1:B:272:VAL:HA	2.18	0.43
1:A:61[B]:ARG:NH1	1:A:61[B]:ARG:HG2	2.33	0.42
1:A:134:LYS:NZ	3:A:1111:HOH:O	2.52	0.42
1:B:213:LEU:HB3	1:B:222[A]:VAL:HG12	2.02	0.41
1:A:61[B]:ARG:N	1:A:61[B]:ARG:HD3	2.36	0.41
1:B:117:GLN:CD	1:B:119[B]:GLU:OE2	2.59	0.41
1:A:107:PHE:CD2	1:A:115:LEU:HD23	2.56	0.41
1:A:118:PHE:HB3	1:A:149:TRP:CZ3	2.56	0.41
1:A:265:SER:O	1:A:272:VAL:HA	2.21	0.41
1:B:127:THR:O	1:B:139:SER:HA	2.21	0.41
1:A:46:PHE:CE1	1:A:298:ALA:HB2	2.56	0.41
1:A:43:LYS:HD3	1:A:85:CYS:HA	2.03	0.40

There are no symmetry-related clashes.

5.3 Torsion angles (i)

5.3.1 Protein backbone (i)

In the following table, the Percentiles column shows the percent Ramachandran outliers of the chain as a percentile score with respect to all X-ray entries followed by that with respect to entries of similar resolution.

The Analysed column shows the number of residues for which the backbone conformation was analysed, and the total number of residues.

Mol	Chain	Analysed	Favoured	Allowed	Outliers	Percentiles	
1	A	303/388 (78%)	293 (97%)	10 (3%)	0	100	100
1	B	303/388 (78%)	290 (96%)	13 (4%)	0	100	100
All	All	606/776 (78%)	583 (96%)	23 (4%)	0	100	100

There are no Ramachandran outliers to report.

5.3.2 Protein sidechains (i)

In the following table, the Percentiles column shows the percent sidechain outliers of the chain as a percentile score with respect to all X-ray entries followed by that with respect to entries of similar resolution.

The Analysed column shows the number of residues for which the sidechain conformation was analysed, and the total number of residues.

Mol	Chain	Analysed	Rotameric	Outliers	Percentiles	
1	A	258/339 (76%)	252 (98%)	6 (2%)	50	37
1	B	257/339 (76%)	253 (98%)	4 (2%)	62	54
All	All	515/678 (76%)	505 (98%)	10 (2%)	62	46

All (10) residues with a non-rotameric sidechain are listed below:

Mol	Chain	Res	Type
1	A	61[A]	ARG
1	A	61[B]	ARG
1	A	213[A]	LEU
1	A	213[B]	LEU
1	A	312	ILE
1	A	318	ARG
1	B	27	ASN
1	B	34	GLN

Continued on next page...

Continued from previous page...

Mol	Chain	Res	Type
1	B	61	ARG
1	B	71	GLU

Sometimes sidechains can be flipped to improve hydrogen bonding and reduce clashes. All (1) such sidechains are listed below:

Mol	Chain	Res	Type
1	B	70	GLN

5.3.3 RNA (i)

There are no RNA molecules in this entry.

5.4 Non-standard residues in protein, DNA, RNA chains (i)

There are no non-standard protein/DNA/RNA residues in this entry.

5.5 Carbohydrates (i)

There are no monosaccharides in this entry.

5.6 Ligand geometry (i)

Of 5 ligands modelled in this entry, 5 are unknown - leaving 0 for Mogul analysis.

There are no bond length outliers.

There are no bond angle outliers.

There are no chirality outliers.

There are no torsion outliers.

There are no ring outliers.

No monomer is involved in short contacts.

5.7 Other polymers (i)

There are no such residues in this entry.

5.8 Polymer linkage issues (i)

There are no chain breaks in this entry.

6 Fit of model and data (i)

6.1 Protein, DNA and RNA chains (i)

In the following table, the column labelled '#RSRZ> 2' contains the number (and percentage) of RSRZ outliers, followed by percent RSRZ outliers for the chain as percentile scores relative to all X-ray entries and entries of similar resolution. The OWAB column contains the minimum, median, 95th percentile and maximum values of the occupancy-weighted average B-factor per residue. The column labelled 'Q< 0.9' lists the number of (and percentage) of residues with an average occupancy less than 0.9.

Mol	Chain	Analysed	<RSRZ>	#RSRZ>2			OWAB(Å^2)	Q<0.9
1	A	301/388 (77%)	0.50	14 (4%)	31	25	8, 14, 29, 41	0
1	B	300/388 (77%)	0.47	11 (3%)	41	36	8, 15, 29, 46	0
All	All	601/776 (77%)	0.49	25 (4%)	36	30	8, 15, 29, 46	0

All (25) RSRZ outliers are listed below:

Mol	Chain	Res	Type	RSRZ
1	A	68	TYR	4.0
1	B	68	TYR	3.9
1	A	248	HIS	3.1
1	A	111	GLY	3.0
1	A	67	ARG	2.9
1	B	114	PRO	2.7
1	A	171	CYS	2.6
1	A	19	ILE	2.6
1	B	171	CYS	2.6
1	B	112	ARG	2.6
1	B	38	PHE	2.5
1	A	112	ARG	2.4
1	B	20	THR	2.4
1	A	93	ARG	2.3
1	B	69	SER	2.2
1	B	115	LEU	2.2
1	B	158	LEU	2.2
1	A	21	GLN	2.2
1	A	114	PRO	2.2
1	A	241	LEU	2.2
1	B	248	HIS	2.1
1	A	117	GLN	2.1
1	A	286	PHE	2.1
1	B	70	GLN	2.1

Continued on next page...

Continued from previous page...

Mol	Chain	Res	Type	RSRZ
1	A	38	PHE	2.1

6.2 Non-standard residues in protein, DNA, RNA chains (i)

There are no non-standard protein/DNA/RNA residues in this entry.

6.3 Carbohydrates (i)

There are no monosaccharides in this entry.

6.4 Ligands (i)

In the following table, the Atoms column lists the number of modelled atoms in the group and the number defined in the chemical component dictionary. The B-factors column lists the minimum, median, 95th percentile and maximum values of B factors of atoms in the group. The column labelled 'Q< 0.9' lists the number of atoms with occupancy less than 0.9.

Mol	Type	Chain	Res	Atoms	RSCC	RSR	B-factors(Å^2)	Q<0.9
2	UNX	A	1001	1/1	0.74	0.77	55,55,55,55	0
2	UNX	B	402	1/1	0.76	0.87	58,58,58,58	0
2	UNX	B	401	1/1	0.77	0.86	51,51,51,51	0
2	UNX	A	1003	1/1	0.83	0.70	43,43,43,43	0
2	UNX	A	1002	1/1	0.90	0.61	50,50,50,50	0

6.5 Other polymers (i)

There are no such residues in this entry.
